# Comprehensive RNA-Sequencing Analysis in Serum and Muscle Reveals Novel Small RNA Signatures with Biomarker Potential for DMD

**DOI:** 10.1016/j.omtn.2018.08.005

**Published:** 2018-08-17

**Authors:** Anna M.L. Coenen-Stass, Helena Sork, Sole Gatto, Caroline Godfrey, Amarjit Bhomra, Kaarel Krjutškov, Jonathan R. Hart, Jakub O. Westholm, Liz O’Donovan, Andreas Roos, Hanns Lochmüller, Pier Lorenzo Puri, Samir EL Andaloussi, Matthew J.A. Wood, Thomas C. Roberts

**Affiliations:** 1 Department of Physiology, Anatomy and Genetics, University of Oxford, South Parks Road, Oxford OX1 3QX, UK; 2 Department of Laboratory Medicine, Karolinska Institutet, Huddinge 141 86, Sweden; 3 Development, Aging and Regeneration Program, Sanford Burnham Prebys Medical Discovery Institute, La Jolla, CA 92037, USA; 4 Department of Biosciences and Nutrition, Center for Innovative Medicine, Karolinska Institutet, Huddinge 141 83, Sweden; 5 Competence Centre on Health Technologies, Tartu 50410, Estonia; 6 Department of Molecular and Experimental Medicine, The Scripps Research Institute, 10550 N. Torrey Pines Road, La Jolla, CA 92037, USA; 7 Science for Life Laboratory, Department of Biochemistry and Biophysics, Stockholm University, 17121 Solna, Sweden; 8 Medical Research Council, Laboratory of Molecular Biology, Francis Crick Avenue, Cambridge CB2 0QH, UK; 9 The John Walton Muscular Dystrophy Research Centre, MRC Centre for Neuromuscular Diseases, Institute of Genetic Medicine, Newcastle University, Central Parkway, Newcastle upon Tyne NE1 3BZ, UK; 10 Biomedical Research Department, Leibniz-Institute für Analytische Wissenschaften-ISAS-e.V., Otto-Hahn-Strasse 6b, 44227 Dortmund, Germany; 11 Department of Neuropediatrics and Muscle Disorders, Medical Center–University of Freiburg, Faculty of Medicine, Freiburg, Germany; 12 Centro Nacional de Análisis Genómico (CNAG-CRG), Center for Genomic Regulation, Barcelona Institute of Science and Technology (BIST), Barcelona, Spain; 13 IRCCS Fondazione Santa Lucia, Rome, Italy

**Keywords:** small RNA sequencing, microRNA, piRNA, Duchenne muscular dystrophy, extracellular miRNA

## Abstract

Extracellular small RNAs (sRNAs), including microRNAs (miRNAs), are promising biomarkers for diseases such as Duchenne muscular dystrophy (DMD), although their biological relevance is largely unknown. To investigate the relationship between intracellular and extracellular sRNA levels on a global scale, we performed sRNA sequencing in four muscle types and serum from wild-type, dystrophic *mdx*, and *mdx* mice in which dystrophin protein expression was restored by exon skipping. Differentially abundant sRNAs were identified in serum (mapping to miRNA, small nuclear RNA [snRNA], and PIWI-interacting RNA [piRNA] loci). One novel candidate biomarker, miR-483, was increased in both *mdx* serum and muscle, and also elevated in DMD patient sera. Dystrophin restoration induced global shifts in miRNA (including miR-483) and snRNA-fragment abundance toward wild-type levels. Specific serum piRNA-like sRNAs also responded to exon skipping therapy. Absolute miRNA expression in muscle was positively correlated with abundance in the circulation, although multiple highly expressed miRNAs in muscle were not elevated in *mdx* serum, suggesting that both passive and selective release mechanisms contribute to serum miRNA levels. In conclusion, this study has revealed new insights into the sRNA biology of dystrophin deficiency and identified novel DMD biomarkers.

## Introduction

Mammalian cells express a plethora of small RNA (sRNA) species, the most extensively studied of which are microRNAs (miRNAs). These ∼22 nt RNA molecules are progressively processed from longer primary miRNA transcripts and subsequently incorporated into Argonaute proteins (e.g., AGO2), where they act to repress the expression of partially complementary transcripts via one of several mechanisms.^1^ As such, miRNAs are key regulators of both physiological and pathophysiological processes. For example, specific muscle-enriched miRNAs (the myomiRs; miR-1a-3p, miR-133a-3p, and miR-206-3p) regulate myoblast proliferation and differentiation during muscle growth and development.^2^, ^3^, ^4^, ^5^, ^6^ Furthermore, a set of miRNAs is differentially expressed in the muscles of Duchenne muscular dystrophy (DMD) patients and dystrophic animal models,^7^, ^8^, ^9^, ^10^ where they contribute to disease-associated processes such as muscle regeneration,^11^, ^12^ inflammation,^13^ fibrosis,^14^ and the regulation of dystrophin expression.^15^

Recently it was shown that miRNAs (and other sRNAs) are present in biofluids (such as serum, plasma, urine, and cerebral spinal fluid).^16^ Importantly, disease-associated changes in biofluid miRNA levels may serve as indicators of underlying tissue pathology^17^ and have utility for monitoring the effectiveness of experimental therapies in clinical trials. Extracellular (ex)-miRNAs have thus been investigated as minimally invasive biomarkers for DMD, an unmet clinical need.^18^, ^19^ As such, myomiRs have been found to be highly enriched in dystrophic serum.^9^, ^20^, ^21^, ^22^ Importantly, therapeutic restoration of dystrophin protein expression by exon skipping (using both virus-encoded U1/U7-small nuclear RNA [snRNA] and non-viral peptide-phosphorodiamidate morpholino oligonucleotide [PPMO] approaches) induced a restoration of circulating ex-myomiRs toward wild-type levels in dystrophic mice.^9^, ^20^, ^22^, ^23^

Building on these promising animal studies, ex-miRNAs were measured in the serum of DMD patients treated with eteplirsen (a naked phosphorodiamidate morpholino oligonucleotide [PMO] antisense oligonucleotide designed to induce skipping of *DMD* exon 51) for 12 weeks, whereby a trend toward therapeutic restoration was observed that did not reach statistical significance.^24^ Importantly, achieving efficient restoration of dystrophin protein and accurate measurement of its expression in human dystrophic muscle remain significant challenges for the field.^25^

The biological and clinical significance of altered extracellular sRNA levels are currently not well understood. Although a number of reports show that circulating miRNAs are capable of mediating intracellular communication, other studies support the notion that these miRNAs are non-functional byproducts of tissue turnover or cellular activity.^26^ In the case of DMD, it has been further assumed that increased permeability of the sarcolemma in dystrophic muscle^27^ results in the passive leakage of cellular contents (including miRNAs) into the circulation.

Our group has previously sought to address these issues in the dystrophin-deficient *mdx* mouse. Expression profiling of key dystrophy-associated miRNAs in multiple muscle groups revealed that in general, differentially expressed miRNAs in dystrophic muscle were not similarly differentially abundant in dystrophic serum,^9^ thereby highlighting that changes in ex-miRNA abundance cannot simply be explained by expression changes in muscle. Similarly, we have recently shown that the elevation of ex-myomiR levels can be a physiological phenomenon that occurs during post-natal muscle development, in the regenerative phase after exercise-induced muscle injury, and concomitant with myoblast differentiation in culture.^28^

Although the majority of extracellular sRNA research effort has been directed toward the study of miRNAs on account of their established role as regulators of gene expression within cells, a number of reports have detected other sRNA species in biofluids.^29^, ^30^, ^31^ Relatively little is known about the biological significance of these non-miRNA sRNA species. For example, tRNA fragments have been shown to exhibit tissue-specific expression patterns^32^ and are associated with Argonaute proteins, suggesting they may be capable of entering the miRNA pathway^33^ or contribute to gene regulation in other ways.^34^, ^35^, ^36^ Similarly, miRNA-like sRNAs derived from snoRNAs and snRNAs have also been reported in cells.^37^, ^38^ Irrespective of their biological functions, or lack thereof, non-miRNA sRNAs constitute a largely overlooked pool of potential serum biomarkers.

Here we have undertaken a comprehensive sequencing study of sRNA expression in serum and four different muscle groups taken from wild-type, dystrophic *mdx*, and exon-skipping-treated *mdx* mice. The aims of these analyses were to: (1) identify novel serum miRNA biomarkers for DMD; (2) analyze differentially expressed miRNAs in dystrophic muscle that may contribute to disease pathophysiology or be therapeutic targets; (3) explore the relationship between global serum and muscle miRNA levels, to investigate the potential selectivity of miRNA export or retention; (4) detect novel (i.e., unannotated) miRNAs expressed in muscle; and (5) investigate the biomarker potential of non-miRNA sRNAs in dystrophic serum and muscle.

## Results

### Study Design

To investigate sRNA levels in dystrophic serum and muscle, we utilized a high-throughput sRNA-sequencing (sRNA-seq) approach in wild-type C57Bl/10 (C57), dystrophic (*mdx*), and PPMO-treated *mdx* mice (all 14-week-old males) (Figure S1A). Successful *Dmd* exon skipping and rescue of dystrophin expression was confirmed by qRT-PCR and western blot, respectively (Figures S1B and S1C). Total RNA was extracted from serum (n = 4) and four different muscles (diaphragm, gastrocnemius, soleus and tibialis anterior [TA]) (n = 2 each). sRNA libraries were generated for each sample, pooled into two 24-plex libraries, and sequencing was performed on the Illumina HiSeq 2500 platform. Sequencing reads were processed using a custom analysis pipeline (Figure S1D). In total, 49 million and 161 million 51 nt single-end reads were generated for the serum and muscle libraries, respectively (Figure S2). In brief, the miRDeep2 package^39^ was used to computationally excise putative miRNA precursors from the mouse genome (mm10) (thus enabling the detection of previously undescribed miRNAs). sRNA reads were mapped to annotated miRNAs from miRBase^40^ or miRDeep2-defined (i.e., empirically determined) precursors, and the number of reads associated with each miRNA counted. In parallel, sequencing reads were aligned to the mouse genome using Bowtie,^41^ and reads mapping to non-coding RNA (ncRNA) species (i.e., miRNA, tRNA, rRNA, mtRNA, snRNA, small nucleolar RNA [snoRNA], small Cajal body-specific RNA [scaRNA], and PIWI-interacting RNA [piRNA]) counted using HTSeq.^42^ Differential expression analysis was performed on counts data generated by either approach using the DESeq package^43^ (Figure S1D; Supplemental Materials and Methods).

### sRNA Analysis in Dystrophic Serum

**Figure 1:**
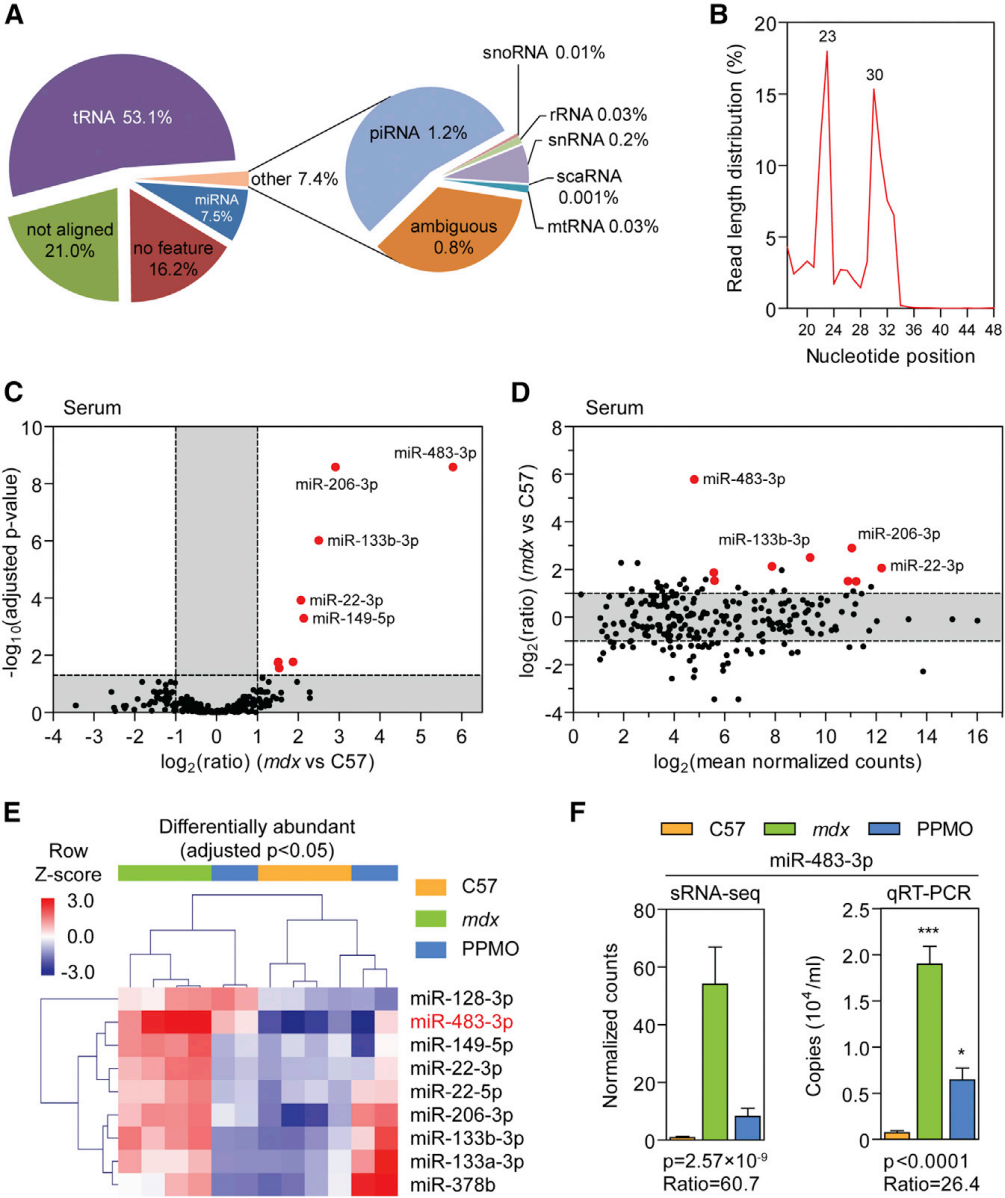
sRNA Analysis in Dystrophic Serum Mapped sRNA reads from serum libraries were sorted into the following ncRNA classes: miRNA, tRNA, rRNA, snRNA, snoRA, scaRNA, mtRNA, and piRNA. (A) Pie chart showing percentage of reads mapping to each ncRNA category for all serum samples. (B) Size distribution of sRNA reads after adaptor trimming in all serum libraries. Differential serum abundance of miRNAs in *mdx* relative to wild-type controls as visualized by (C) volcano plot and (D) MA plot. Statistically significant changes are highlighted in red and blue (for elevated and reduced levels in *mdx* serum, respectively). Labels are shown for miRNAs of interest. (E) Heatmap of significantly changed miRNAs in *mdx* serum, showing the effect of PPMO treatment on circulating miRNA levels. The label for miR-483-3p is highlighted in red. Scale bars show mean-centered log_2_ normalized counts (row *Z* score), where red and blue indicate higher and lower than mean abundance, respectively. (F) Serum abundance data for miR-483-3p determined by sRNA-seq and sRNA qRT-PCR. All values are mean + SEM; n = 4. p values are calculated by negative binomial distribution test (with Benjamini-Hochberg correction for multiple comparisons) for sRNA-seq or one-way ANOVA for qRT-PCR. *mdx* versus C57 fold change are indicated, *p < 0.05; ***p < 0.001 (Bonferroni *post hoc* test).

Analysis of serum sRNA alignments revealed that the majority (∼53%) of reads mapped to tRNA genes. In contrast, only ∼8% of reads mapped to miRNAs, and ∼1% to piRNAs. Other annotated small ncRNA species comprised less than 1% of the total reads (Figure 1A). The sRNA proportions were largely similar between experimental groups, although the miRNA fraction was slightly larger in the *mdx* samples (Figure S3A). Sequence read lengths exhibited a bimodal distribution after adaptor removal, with peaks at 23 and 30 nt in length (Figure 1B). Given the high percentage of reads mapping to tRNAs, these peaks likely represent two different populations of tRNA fragments. Analysis of the miRNA-mapping reads revealed 956 distinct annotated miRNA species detected across all serum-derived libraries. However, the distribution of reads was highly uneven, with 79 miRNAs comprising 99% of all reads and the most abundant miRNA (miR-1a-3p) accounting for 68% of all miRNA-mapping reads (Figures S3B and S3C). Differentially abundant miRNAs in *mdx* serum were visualized by volcano and MA plots (Figures 1C and 1D). The levels of nine miRNAs were significantly upregulated in *mdx* serum at the p < 0.05 level (adjusted for multiple comparisons using the Benjamini-Hochberg method). Heatmap visualization of normalized counts data for statistically changed miRNAs revealed that PPMO treatment in *mdx* mice induced a shift in serum miRNA abundance toward wild-type levels (Figure 1E). One miRNA with no previously known association with dystrophic pathology, miR-483-3p, was the most upregulated (∼60-fold) and one of the most statistically significantly changed miRNAs in *mdx* serum (adjusted p = 2.57 × 10^−9^). Furthermore, miR-483-3p was restored toward wild-type levels in PPMO-treated animals as confirmed by sRNA TaqMan qRT-PCR (Figure 1F).

Consistent with other reports, miR-22-3p, miR-133a-3p, miR-133b-3p, miR-206-3p, and miR-378b levels were also increased in *mdx* serum.^9^, ^20^, ^21^, ^22^, ^44^ miR-1a-3p was not found to be elevated in *mdx* serum, which was surprising because it is one of the three myomiRs (miR-1, miR-133, and miR-206) that have most frequently been reported to be elevated in the dystrophic condition.^9^, ^20^, ^21^, ^22^, ^23^, ^28^, ^44^ qRT-PCR validation confirmed that all three myomiRs (including miR-1a-3p) were significantly elevated (p < 0.0273) in *mdx* serum and restored toward wild-type levels in the PPMO-treated animals (Figure S4). An unrelated control miRNA (miR-126a-3p) was unchanged between experimental groups (Figure S4). These data suggest that the sequencing result for miR-1a-3p constitutes a false negative, whereas results for miR-133a-3p, miR-206-3p, and miR-126a-3p were consistent between both sRNA-seq and qRT-PCR methodologies.

The elevation of two further miRNAs (miR-128-3p and miR-149-5p) in dystrophic serum has, to our knowledge, not been reported previously. miR-149-5p is particularly interesting given that its abundance was almost completely restored to wild-type levels after PPMO treatment (Figure 1E). Our group,^22^ and others,^44^ have previously identified miR-22 as being upregulated in *mdx* and *mdx*^*4CV*^ mouse serum, respectively. In the present study we observed that both major (miR-22-3p) and minor (miR-22-5p) species derived from the miR-22 precursor were elevated in *mdx* serum, despite the latter being ∼100 less abundant than the former. Additionally, both miR-22-derived miRNAs were restored following exon skipping therapy.

### sRNA Analysis in Dystrophic Muscle

**Figure 2:**
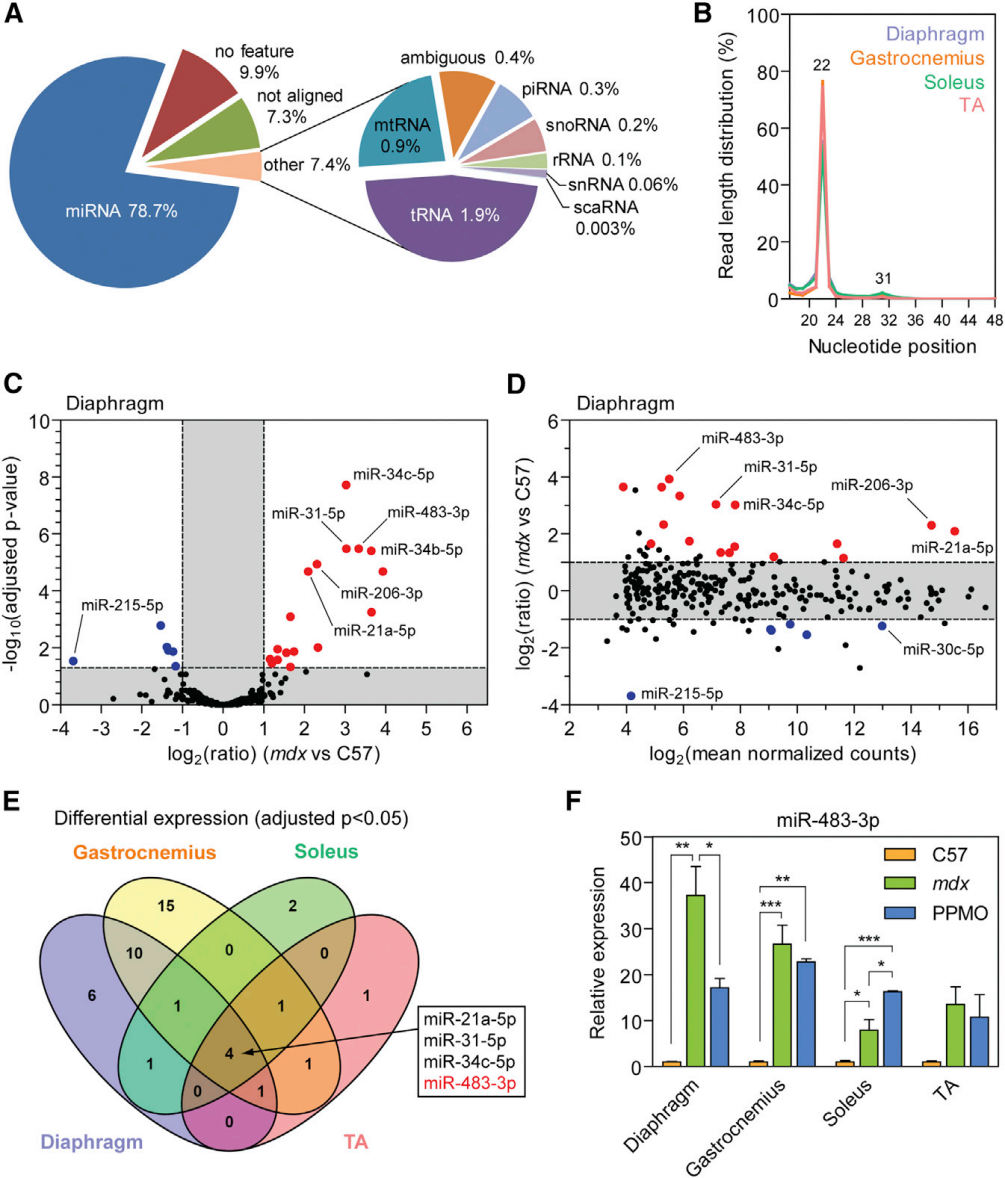
sRNA Analysis in Dystrophic Muscle Mapped sRNA reads from muscle libraries were sorted into the following ncRNA classes: miRNA, tRNA, rRNA, snRNA, snoRA, scaRNA, mtRNA, and piRNA. (A) Pie chart showing percentage of reads mapping to each ncRNA category averaged across all muscle samples (pie charts for each individual muscle are shown in Figure S5). (B) Size distribution of sRNA reads after adaptor trimming in each set of muscle libraries. Differential expression of miRNAs in *mdx* diaphragm relative to wild-type controls as visualized by (C) volcano plot and (D) MA plot (volcano and MA plots for gastrocnemius, soleus, and TA muscles are shown in Figure S8). Statistically significant changes are highlighted in red and blue (for elevated and reduced levels in *mdx* serum, respectively). Labels are shown for miRNAs of interest. (E) Venn diagram showing overlap between differentially expressed miRNAs in dystrophic muscles. miRNAs that were commonly differentially expressed in all four muscle types are indicated. (F) Expression of miR-483-3p was validated by sRNA TaqMan qRT-PCR in each muscle using miR-16-5p as a control for normalization. Values are mean + SEM; n = 3. *p < 0.05; **p < 0.01; ***p < 0.001, one-way ANOVA with Bonferroni *post hoc* test.

In contrast with the serum samples, the muscle-derived libraries contained primarily miRNA-mapping reads (∼79%), whereas only ∼2% and 0.3% mapped to tRNAs and piRNAs, respectively (Figure 2A). In all muscles analyzed, the distribution of read lengths post-adaptor removal showed a prominent peak at 22 nt, consistent with the expected length of miRNAs (Figure 2B). Analysis of ncRNA classes across each individual muscle group revealed the same general pattern, although the gastrocnemius libraries were more enriched for miRNA reads (∼93%), and the soleus libraries contained a greater proportion of tRNA-mapping reads (4%) (Figure S5). The distribution of ncRNA-mapping reads was similar between experimental animal groups, although the number of miRNA-mapping reads was generally greater in the *mdx* group for each muscle (Figure S6).

As with the serum samples, the miRNA composition of the muscle libraries was highly uneven. The total number of miRNAs detected ranged from 792 in gastrocnemius to 920 in diaphragm, although less than 80 miRNAs made up 99% of all miRNA reads (Figures S7A and S7B). In line with the serum data, miR-1a-3p was the most abundant species in each muscle. Comparison of the top 20 highest abundance miRNAs revealed that 18 miRNAs were common between all muscles, suggesting that the overall composition of the miRNome is largely similar between different muscle types (Figures S7C and S7D). These library characteristics are consistent with other skeletal muscle sRNA-seq studies using the Illumina platform.^45^, ^46^

Differentially expressed miRNAs in *mdx* diaphragm were visualized by volcano plot (Figure 2C) and MA plot (Figure 2D; data for gastrocnemius, soleus, and TA are shown in Figure S8), and miR-483-3p was found to be upregulated in all four *mdx* muscles (Figure 2E). Furthermore, miR-483-3p sequencing data showed a restoration toward wild-type levels in both diaphragm and gastrocnemius muscles (Figure S10), and this finding was further validated by qRT-PCR (Figure 2F). Three other miRNAs, miR-21a-5p, miR-31-5p, and miR-34c-5p, were commonly upregulated in all muscles tested (Figure 2E). Notably, miR-206-3p was significantly elevated in diaphragm and gastrocnemius, and upregulated but not significant in TA. Many miRNAs were found to be uniquely differentially expressed in a single muscle, underlining the complexity of the miRNA transcriptome in dystrophic muscles (differential miRNA expression data are described in full in Data S1).

Previously we have shown that exon skipping with a different PPMO conjugate (Pip6e-PMO) resulted in widespread shifts in protein and mRNA expression toward wild-type levels in dystrophic TA, whereas the miRNome was largely unaffected.^10^ We reasoned that a higher level of dystrophin restoration might be required to induce a global shift in the dystrophic muscle miRNome, and so in the present study, *mdx* mice were treated with a more potent PPMO conjugate (Pip6a-PMO)^22^, ^47^ (6%–40% dystrophin protein restoration; Figure S1). Therapeutic restoration of global miRNA expression was assessed in each muscle type by principal component analysis and hierarchical clustering of significantly differentially expressed miRNAs (Figures S9 and S10). Partial shifts in the miRNome toward wild-type levels were observed in the diaphragm, gastrocnemius, and soleus. However, consistent with our previous study,^10^ little or no therapeutic restoration was observed in PPMO-treated TA muscles. These findings were further supported by qRT-PCR validation of miR-21a-5p, miR-31-5p, miR-34c-5p, and miR-206-3p expression, which exhibited similar responses to therapy in diaphragm and gastrocnemius, but not in soleus or TA (Figure S11). These data are indicative of a heterogeneous response between muscles after exon skipping therapy and further highlight that miRNA fold changes between serum and muscle are not necessarily correlated.

To account for the limited sample size in the muscle comparisons, we performed an additional differential expression analysis with all muscles pooled together such that there were n = 8 subjects in each experimental group. Although this analysis is unable to detect differentially expressed miRNAs that are specific to a particular muscle type, the increased statistical power potentially enables the detection of further miRNAs that are commonly differentially expressed in the dystrophic condition (Figure S12). The C57 samples were found to cluster together, whereas the *mdx* and PPMO-treated samples were intermingled (Figure S12C), consistent with the variable response of the muscle miRNome to exon skipping described above (Figures S9–S11). 33 miRNAs were determined to be differentially expressed (adjusted p < 0.05) in the pooled analysis, which included the common miRNA signature described above (Figure 2E). 20 of these miRNAs were differentially expressed in at least one muscle group. The remaining 13 miRNAs were typically very lowly abundant (e.g., miR-483-5p, the minor form of miR-483-3p).

### Relationship between miRNA Levels in Serum and Muscle

**Figure 3:**
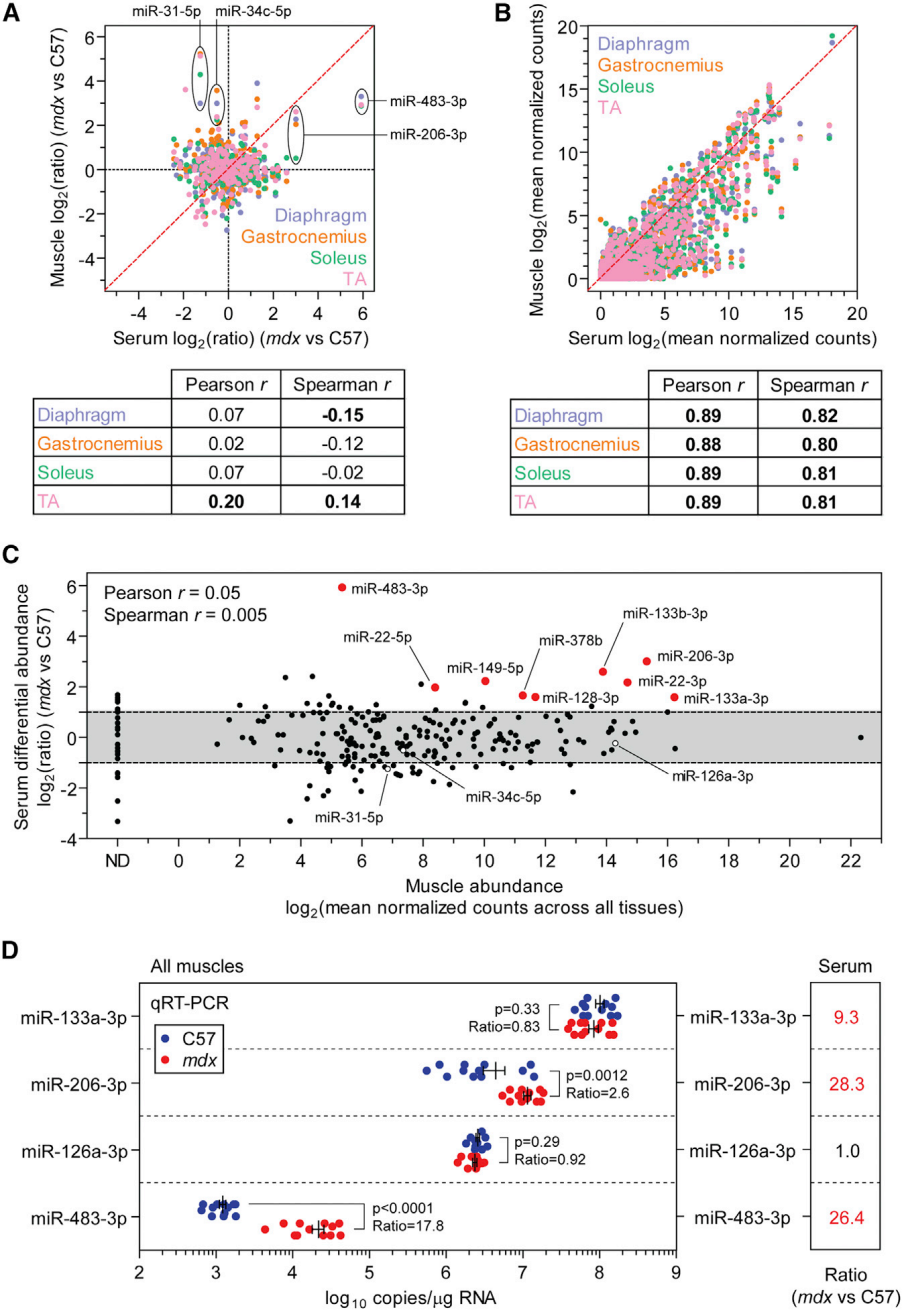
Relationship between Serum and Muscle miRNA Levels Scatterplots of (A) *mdx* versus C57 miRNA relative expression ratios, and (B) miRNA absolute abundance (i.e., mean normalized counts), comparing serum against each muscle. Pearson and Spearman coefficients are indicated, and statistically significant correlations (p < 0.05) are highlighted in bold. The hypothetical line of perfect correlation is shown in red. Labels indicate miRNAs of interest. (C) Plot of relative serum abundance ratios (*mdx* versus C57) against absolute miRNA abundance in muscle (i.e., normalized counts averaged across all muscles). Significantly elevated and lowered miRNAs (adjusted p < 0.05) are highlighted in red and blue, respectively. Other miRNAs of interest are highlighted with open circles. (D) Absolute copy numbers were determined by qRT-PCR to validate four illustrative examples: miR-133a-3p, miR-206-3p, miR-126a-3p, and miR-483-3p, in all C57 and *mdx* muscle samples (n = 12). Serum *mdx* versus C57 expression ratios are shown in the right panel, and statistically significant (adjusted p < 0.0001; n = 4) changes are highlighted in red (data are taken from Figures 1F and S4). Values are mean ± SEM. ND, not detected in muscle.

Ex-miRNAs may enter the circulation as a consequence of: (1) passive leakage from necrotic, damaged, or transiently permeable muscle; (2) controlled release; or (3) a combination of both processes. Previously we found that there was little relationship between the relative miRNA changes observed in muscle and the corresponding expression ratio changes in serum samples taken from the same animals, based on a focused analysis of 11 dystrophy-associated miRNAs.^9^ To further investigate this phenomenon on a global scale, we compared relative miRNA levels between the muscle and serum in the sRNA-seq libraries. Consistent with our previous observations, there was no correlation, or only a very weak correlation, between serum and muscle *mdx* versus C57 expression ratios (Figure 3A). Two notable exceptions were miR-483-3p and miR-206-3p, which were increased in both serum and muscle. Conversely, two of the most upregulated miRNAs in *mdx* muscle (miR-31-5p and miR-34c-5p) were present at reduced levels in *mdx* serum relative to C57 controls. These data show that an increase in tissue miRNA expression does not result in elevated levels of serum miRNAs in the majority of cases. In contrast, miRNA counts in serum were strongly positively correlated with counts data from each muscle (Pearson *r* > 0.883, Spearman *r* > 0.802, p < 0.0001) (Figure 3B), indicating that the level of miRNA expression in muscle is generally reflected in its overall abundance in serum (here we assume that, in the general case, the number of read counts is correlated with absolute abundance, although this may not be true in some specific cases due to biases in library preparation.)

Based on these findings, we next investigated whether absolute muscle expression levels can account for the relative changes in ex-miRNA abundance observed in serum. Specifically, if miRNAs are passively released from dystrophic muscle, then the most abundant miRNAs in muscle would be expected to be the most differentially changed species in *mdx* serum. In this scenario, ex-miRNAs might be considered to be nuclease-stable, cellular waste that accumulates in the circulation due to an increase in membrane permeability in dystrophic muscle and/or myofiber death. To test this hypothesis, we plotted relative serum abundance (i.e., *mdx* versus C57 expression ratios) against absolute expression in muscle (i.e., normalized counts averaged across all muscle libraries) (Figure 3C). No correlation was found between these parameters. Differentially abundant serum miRNAs were detected over a wide range of absolute muscle expression levels, and many highly abundant miRNAs in muscle were not differentially abundant in *mdx* serum, thereby demonstrating that tissue absolute abundance is not the only determinant of changes in ex-miRNA levels in the dystrophic condition. Similar results were obtained when the sRNA-seq data for each muscle or experimental group were considered separately (data not shown). Absolute quantification qRT-PCR validation revealed four illustrative scenarios (Figure 3D). First, the myomiR miR-133a-3p is present at very high overall levels in muscle, is unchanged in *mdx* muscle, and is elevated in *mdx* serum. This example demonstrates that changes in tissue expression and serum abundance are not necessarily coupled. Second, another myomiR, miR-206-3p, is expressed at high levels in muscle (although ∼10-fold lower than that observed for miR-133a-3p) and is significantly elevated in the *mdx* muscles (diaphragm, gastrocnemius, and TA) and in serum. miR-206-3p is known to be enriched in regenerating myofibers,^7^, ^11^, ^12^ and so increases in both tissue and serum levels likely reflect the regenerative status of dystrophic muscle. Third, in contrast, the non-myomiR miR-126a-3p is expressed at similar levels to miR-206-3p but is unchanged in either muscle or serum, thereby indicating that some highly expressed miRNAs are selectively retained, contrary to what would be expected if ex-miRNAs passively leak from damaged muscle. Fourth, lastly, miR-483-3p is elevated in all four *mdx* muscle and serum, but in contrast with the myomiRs, is present at very low levels in muscle (∼1,000–100,000-fold fewer reads than for miR-133a-3p). miR-483-3p therefore demonstrates that high tissue expression is not a pre-requisite for increased release during muscle pathology, although it is possible that this miRNA may also originate from other non-muscle tissues. Very similar results were obtained when qRT-PCR data were considered separately for each muscle (data not shown).

### miR-483-5p Is Elevated in DMD Patient Serum

**Figure 4:**
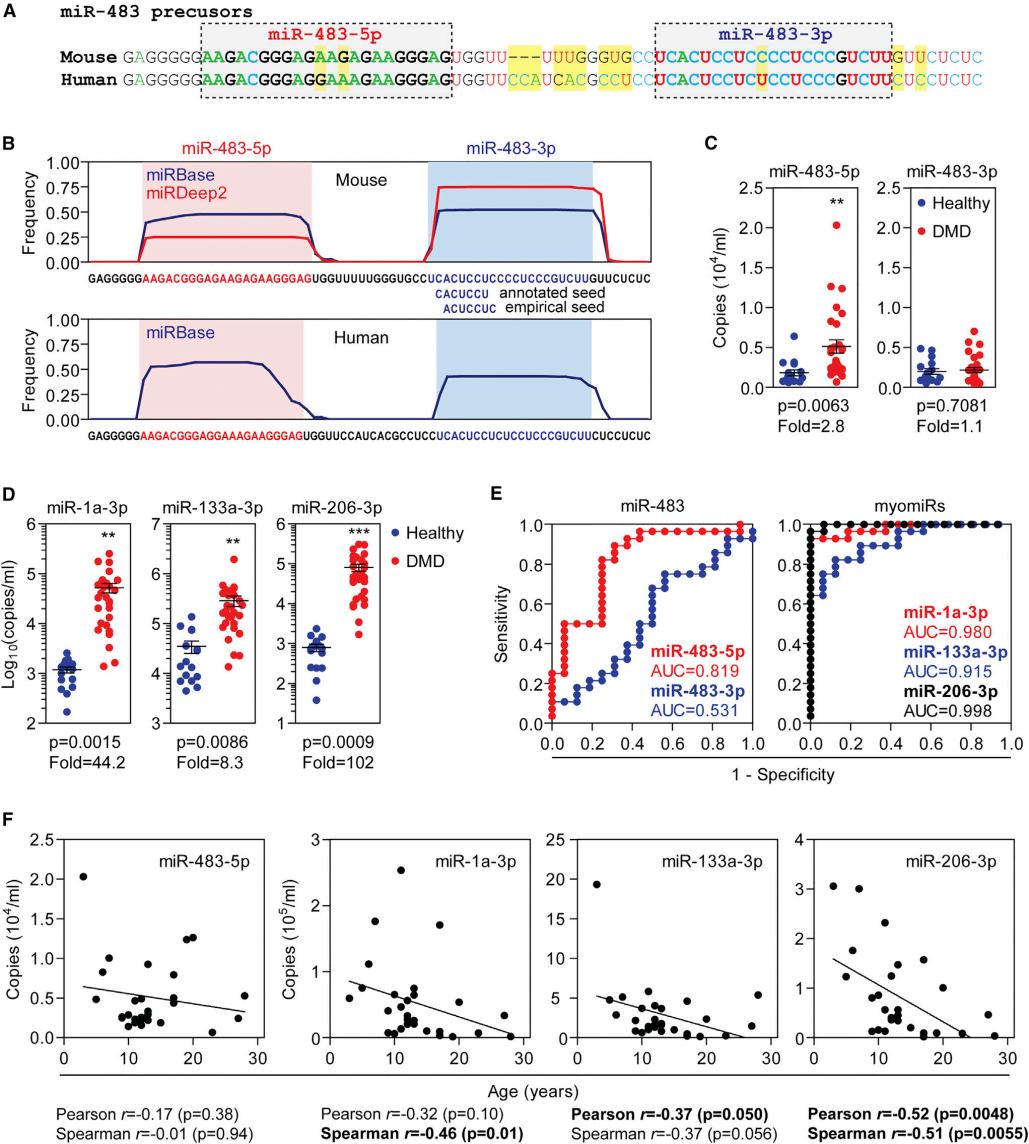
Analysis of miR-483 in DMD Patient Serum (A) Alignment of mouse and human miR-483 precursors. Differences are highlighted in yellow. (B) miRNA signature plots for mouse and human miR-483. Pooled mouse muscle library data are shown in red, and publically available miRBase data are shown in blue. Annotated and empirically derived miRNA seed regions are indicated. Serum from DMD patients (n = 28) and healthy controls (n = 16) were analyzed by sRNA TaqMan qRT-PCR for (C) miR-483-5p and miR-483-3p, and (D) the myomiRs: miR-1a-3p, miR-133a-3p, and miR-206-3p. Individual data points are shown and the mean ± SEM indicated. **p < 0.01; ***p < 0.001, t test. (E) ROC curves for miR-483 and myomiRs. (F) Correlations between miRNA levels and patient age in DMD samples, and statistically significant correlations (p < 0.05) are highlighted in bold. AUC, area under the curve.

Given that miR-483-3p was the most differentially expressed miRNA in *mdx* serum, and highly upregulated in *mdx* muscle, we sought to measure the abundance of this novel candidate biomarker in DMD patient serum. The miR-483 precursor hairpin resides within an intron of the gene encoding the IGF2 (Insulin-like Growth Factor 2) protein in both humans and mice. However, the miR-483 precursor is not completely conserved between species, with non-seed polymorphisms in both 5′ and 3′ arms, and substantial differences in the loop region and 3′ flanking sequence (Figure 4A). Analysis of miRNA signature plots from publicly available miRBase data^40^ and our experimental libraries demonstrated a species-specific difference in strand selection preference, with the 3′ strand favored in mouse and the 5′ strand favored in human (Figure 4B). Additionally, we observed that for the 3′ arm, the sequencing data show that the most prevalent miRNA species is one nucleotide offset relative to the canonical sequence, suggesting that the canonical sequence may have been misannotated (Figure 4B). Consequently, we selected both miR-483-3p and miR-483-5p for further analysis in DMD patient serum (n = 28) and healthy controls (n = 16). miR-483-5p was significantly (p = 0.0063) elevated in DMD serum by 2.8-fold, whereas miR-483-3p was unchanged (Figure 4C). Consistent with previous reports,^20^, ^24^ the myomiRs (miR-1a-3p, miR-133a-3p, and miR-206-3p) were highly elevated in DMD patient serum (∼8–100-fold; p < 0.0086) (Figure 4D). Receiver operating characteristic (ROC) curve analysis showed that miR-483-5p was effective at discriminating between healthy individuals and DMD patients (area under the curve [AUC] = 0.82) (Figure 4E). However, the predictive power of miR-483-5p was weaker than for the myomiRs (AUC ≥ 0.92). Notably, negative correlations were observed between serum myomiR levels and DMD patient age, whereas this effect was less pronounced (and was not significant) for miR-483-5p (Figure 4F). As a result, miR-483-5p may offer an advantage over the myomiRs as pharmacodynamic serum biomarkers, especially in longitudinal studies. No significant correlations between age and serum miRNA levels were observed in healthy individuals for any of the miRNAs assayed (data not shown).

There was little overlap in predicted human mRNA targets for miR-483-5p or miR-483-3p (both canonical and empirically determined seed sequence variants) (Figure S13A) (Data S2). Similarly, gene list enrichment analysis suggested that the two arms of pre-miR-483 likely execute distinct functions (Figure S13B). Given that the strand preference of pre-miR-483 is not conserved between species, these findings suggest that elevation of miR-483 is unlikely to contribute to DMD pathophysiology. Instead, elevated miR-483 may reflect the transcriptional activity of its host gene (*IGF2*) in muscle, because this gene has been reported to be upregulated in *mdx* muscle^10^, ^48^, ^49^ and in DMD patient muscle.^50^ As such, serum miR-483 levels may serve as a minimally invasive means of determining *IGF2* expression in muscle.

### Identification of Novel miRNAs in Muscle

The miRDeep2 algorithm uses a data-driven approach to identify putative miRNA hairpins from sRNA-seq libraries and is therefore capable of detecting previously unannotated miRNAs.^39^ To this end, miRDeep2 output was filtered as described in the Supplemental Materials and Methods, four novel miRNAs were selected for further analysis (Figures S14A–S14D; Table S1) and given the designation “nmm” (novel muscle miRNA) followed by the number of their chromosome of origin (e.g., nmm-1). Minimum free energy values for these novel miRNA structures were ≤−19.8 kcal/mol, suggesting that they all form stable precursor hairpins. Notably, two of the candidate miRNAs had identical seed sequences to miRNAs found in other species (i.e., hsa-miR-5002-3p for nmm-1 and rno-miR-336-3p for nmm-14) (Figure S15A). Gene list enrichment analysis of predicted target transcripts (generated using miRDB;^51^ Data S3) for the novel miRNAs identified enriched Gene Ontology (GO) terms for targets of nmm-1 (i.e., transcription factor DNA binding, steroid hormone receptor activity, and phosphatase complex) and nmm-14 (i.e., smooth muscle cell proliferation) (Figure S15B). No significant GO terms were associated with predicted targets for nmm-16 or nmm-19. Genomic locations of novel miRNA precursors are shown in Figure S16. All four novel miRNAs were detected by qRT-PCR in all muscle samples, although they were found to be lowly abundant (consistent with their sequencing read counts) (Figure S14E). Differences in expression between experimental animals and muscle groups were minimal (Figures S17A–S17D), suggesting that these novel miRNAs are unlikely to be associated with dystrophic pathophysiology.

### Non-miRNA sRNA Analysis in Serum and Muscle

**Figure 5:**
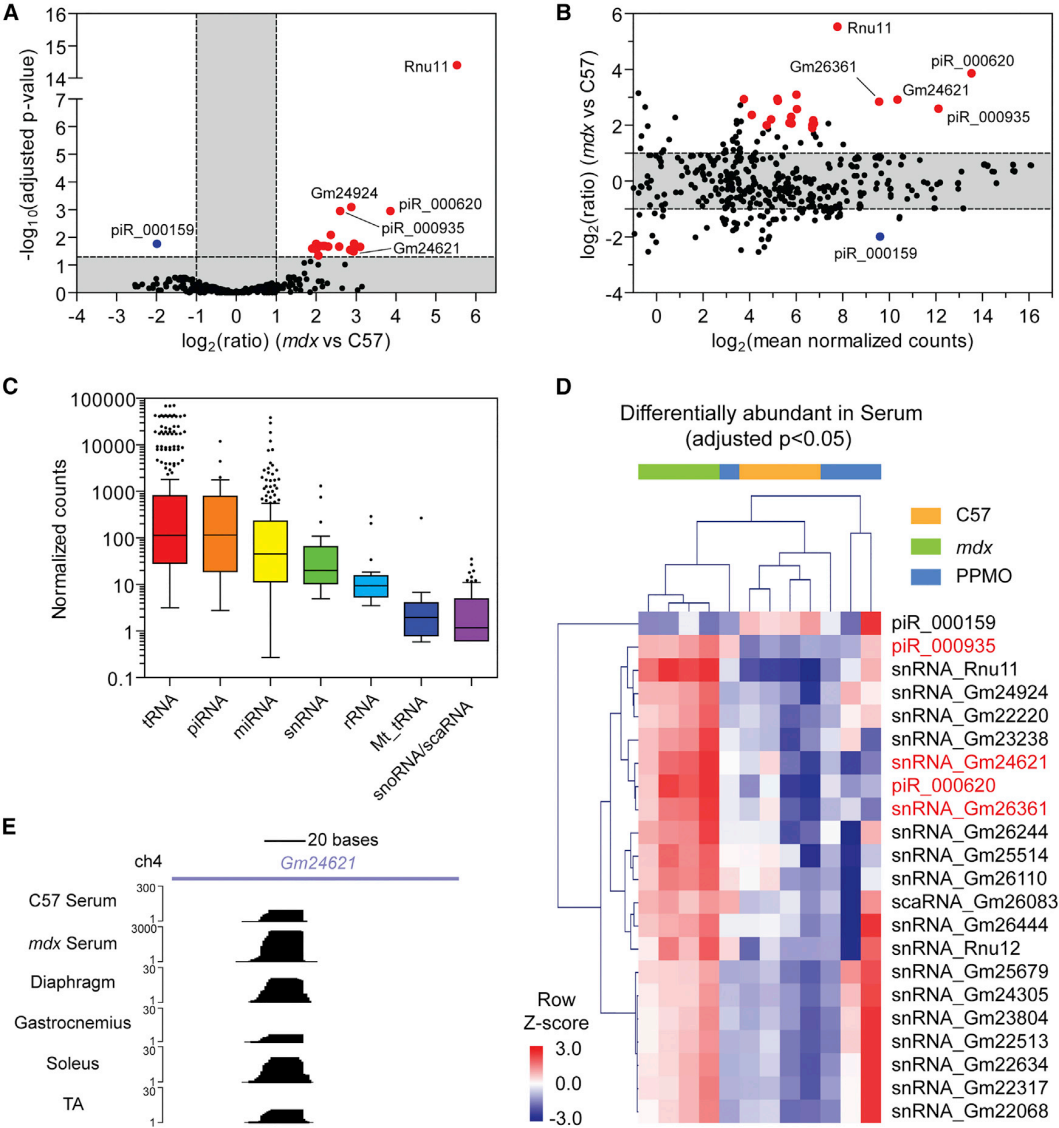
Differential ncRNA Expression in Dystrophic Serum Differential serum abundance of ncRNA-mapping sRNAs in *mdx* relative to C57 (wild-type) (n = 4) controls as visualized by (A) volcano plot and (B) MA plot. Statistically significant (adjusted p < 0.05) changes are highlighted in red and blue (for elevated and reduced levels in *mdx* serum, respectively). Labels are shown for ncRNAs of interest. (C) Tukey boxplot of mean normalized counts values (across all samples, n = 12) for each ncRNA class. (D) Heatmap of significantly changed ncRNAs in *mdx* serum, showing the effect of PPMO treatment on circulating ncRNA levels. ncRNAs of particular interest are highlighted in red. Scale bars show mean-centered, log_2_ normalized counts (row *Z* score), where red and blue indicate higher and lower than mean abundance, respectively. (E) Representative read density plots are shown for the snRNA *Gm24621.*

To investigate the possibility that non-miRNA sRNAs might be biomarkers of dystrophic pathology, we performed differential expression analysis for reads that mapped to other small ncRNA loci. 22 ncRNA-mapping species (18 snRNAs, 1 scaRNA, and 3 piRNAs) were found to be significantly changed (adjusted p < 0.05) in dystrophic serum (Figures 5A and 5B) (differential ncRNA expression data are described in full in Data S4). Analysis of the mean abundance for individual sRNA species contained within each class indicated that the absolute serum abundance of tRNAs, snRNAs, and piRNAs was comparable with that of the miRNAs, whereas sRNAs belonging to the other classes were generally less abundant (Figure 5C). Exon skipping therapy induced a shift in the mean abundance levels toward wild-type levels in the case for many of these differentially abundant non-miRNA sRNAs (Figure 5D). In contrast with serum, few non-miRNA sRNAs were differentially expressed in muscle, and there was little overlap in differentially expressed sRNAs between the different muscle groups (Figures S18 and S19).

We next visually inspected sequencing data for both serum and muscle libraries to identify sRNAs of particular interest. The snRNA genes *Gm24621* and *Gm26361* were both elevated in *mdx* serum by ∼7-fold (p < 0.00102) and exhibited similar patterns of read density: a single peak mapping internally within the primary snRNA transcript with a defined 3′ terminus and a more variable 5′ terminus (e.g., *Gm24621* is shown in Figure 5E). The majority of reads were of 18 nt in length, and a range of minor species was observed with length up to ∼26 nt. Notably, equivalent sequencing peaks were observed in the muscle libraries for both snRNA-derived species, suggesting that the processing of these sRNAs occurs prior to their export to the extracellular space.

**Figure 6:**
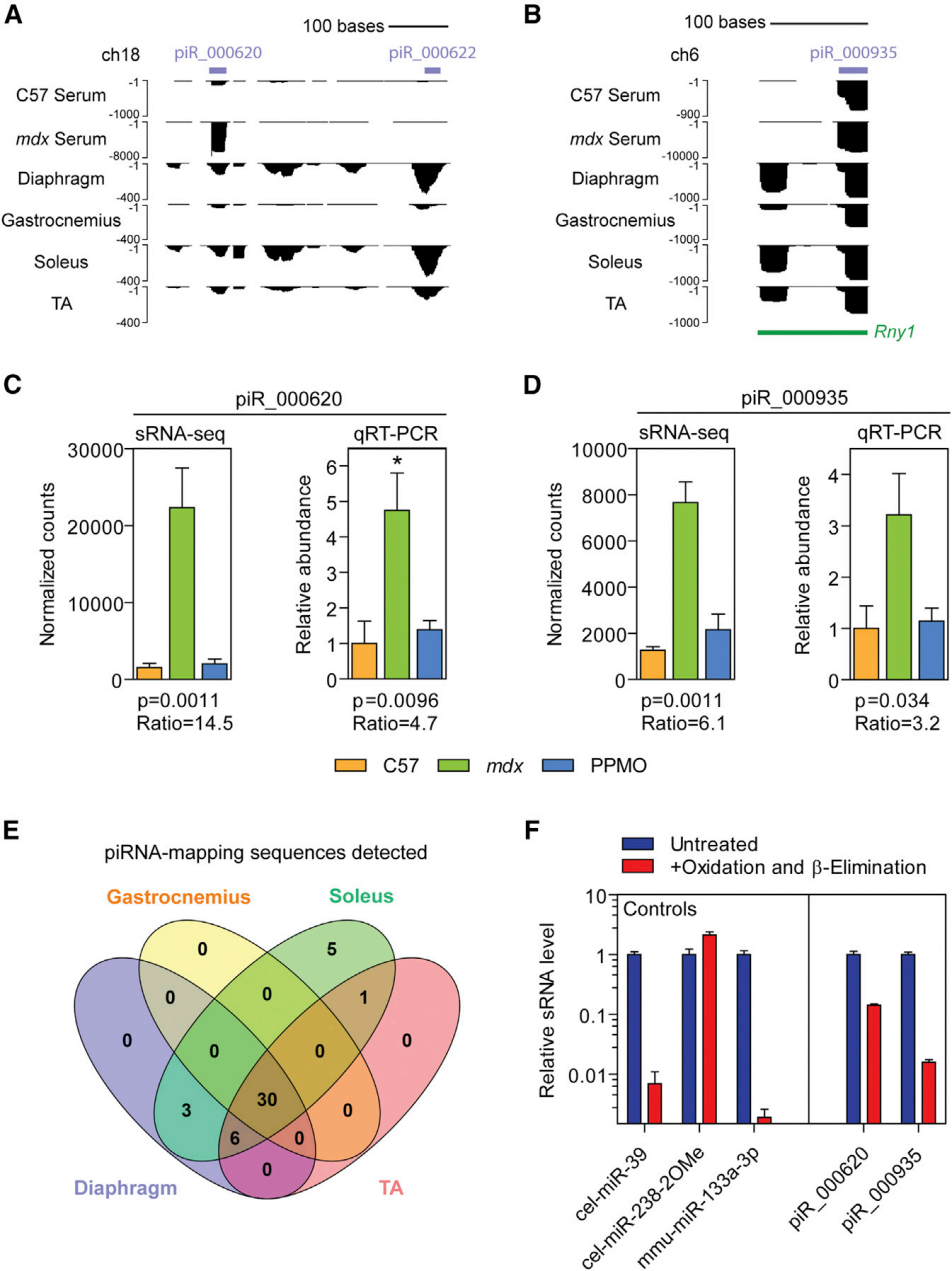
Analysis of piRNA-Mapping sRNA Reads Representative read density plots are shown for C57 and *mdx* serum libraries at the (A) piRNA_000620 and (B) piRNA_000935 loci. Serum piRNA levels were validated in C57, *mdx*, and PPMO-treated *mdx* samples (n = 4) for (C) piRNA 000620 and (D) piRNA 000935. p values represent negative binomial distribution test (adjusted with the Benjamini-Hochberg method) or one-way ANOVA for sRNA-seq and qRT-PCR, respectively. *mdx* versus C57 fold change are indicated, *p < 0.05 (Bonferroni *post hoc* test). Statistical comparisons are with the C57 control. (E) Venn diagram showing commonly detected piRNAs in diaphragm, gastrocnemius, soleus, and TA muscle samples. (F) RNA samples from *mdx* TA muscle were subjected to oxidation and β-elimination, or left untreated, and putative piRNAs analyzed by oligonucleotide linker qRT-PCR (n = 3). An exogenous synthetic oligonucleotide (cel-miR-39) and endogenous miR-133a-3p were used as negative (miRNA-like) controls. A further exogenous synthetic oligonucleotide with a 3′ terminal 2′-O-methyl modification (cel-miR-238-2OMe) was used as a positive (piRNA-like) control. Values are mean + SEM.

piRNAs are of particular interest because these sRNAs have established gene regulatory functions. piRNAs are generated by processes that are distinct from miRNA biogenesis^52^ and so have different biochemical properties. Specifically, piRNAs are longer than miRNAs (24–36 nt) and have 2′-O-methylation (2OMe) ribose modifications at their 3′ termini.^53^ Two differentially abundant piRNAs (piR_000620 and piR_000935) were selected for further study because they were among the most statistically elevated sRNA species in *mdx* serum, were highly abundant overall, and were restored toward wild-type levels by PPMO treatment (Figures 5A and 5B). Inspection of sequencing read density showed that the piRNA-mapping sRNAs were of the approximate expected lengths but with relatively poorly defined termini (Figures 6A and 6B). Notably, other unannotated sRNA species of varying lengths were also found to map at the same loci. Elevation of these two piRNAs in dystrophic serum, and their restoration toward wild-type levels in response to *Dmd* exon skipping, was validated by qRT-PCR (Figures 6C and 6D).

piRNAs are enriched in the gonads (and specifically spermatocytes in mammals), where their primary function is generally accepted to be the defense of genome integrity by suppression of selfish mobile DNA elements (i.e., transposons).^54^, ^55^ However, piRNAs have also been reported to be expressed in other tissues, where their biological significance is less clear.^46^, ^56^ We therefore sought to determine whether the differentially abundant serum piRNAs could originate from muscle. In total, 35 piRNAs were detected in serum, all of which were also detected in at least one muscle. Of the 45 piRNAs detected across all muscles analyzed, 30 piRNAs (including piR_000620 and piR_000935) were present in all four tissues, suggestive of a common muscle signature of piRNA-mapping sRNAs (Figure 6E).

To determine whether the sRNA species detected here are bona fide piRNAs, we performed an assay to determine the chemical identity of their 3′ termini based on an oxidation and β-elimination reaction. This reaction converts the unmodified 2′ hydroxyl groups of the ribose sugar to a di-aldehyde group, which inhibits the ligation of an oligonucleotide linker used for sRNA detection by qRT-PCR. As a result, unmodified sRNAs (i.e., miRNAs) become invisible to qRT-PCR after oxidation and β-elimination, whereas piRNAs are protected from conversion as a consequence of their terminal 2′-O-methylation (Supplemental Materials and Methods). Endogenous (miR-133a-3p) and synthetic exogenous (cel-miR-39) negative controls were utilized, which showed a loss of qPCR signal after oxidation and β-elimination. In contrast, an exogenous positive control oligonucleotide synthesized with a 2OMe modification to mimic the chemistry of piRNAs (cel-miR-238-2OMe) was unaffected. piRNAs of interest were analyzed in total RNA samples taken from *mdx* TA muscles, whereby oxidation and β-elimination resulted in a reduction in qPCR signal of 86% and 98% for piR_000620 and piR_000935, respectively, suggesting that they are largely unmodified at their 3′ termini (Figure 6F). As such, these species should more properly be considered “piRNA-like” sRNAs. Interestingly, piR_000935 also overlapped with the *Rny1* gene (Ro-associated Y1), suggesting that this sRNA may be a 5′ terminal Y RNA fragment (Figure 6B).

## Discussion

Here we have undertaken a comprehensive analysis of small ncRNA levels in dystrophic muscle and serum by high-throughput sequencing of sRNA. Using this approach, we analyzed the relative contributions of various ncRNAs to the composition of the sRNA transcriptome. While miRNAs comprised the majority of sRNAs in the muscle libraries, the serum libraries were much more heterogeneous, with tRNA-mapping fragments being the most prevalent sRNA class (Figures 1, 2, S3, S5, and S6). Additionally, a plethora of sRNAs mapping to snRNA, snoRNA, and piRNA loci were detected in both muscle and serum libraries.

Initially, we focused on ex-miRNAs given their importance in gene regulation within cells and the intense interest surrounding their use as biomarkers in biofluids. As such, a set of differentially abundant serum miRNAs was identified, which included several that were consistent with previous reports from our group and others.^9^, ^20^, ^21^, ^22^, ^44^ One miRNA in particular, miR-483-3p, is a novel DMD candidate biomarker that is elevated in both *mdx* serum and muscle, and was restored toward wild-type levels in serum and diaphragm after PPMO treatment (Figures 1 and 2). In humans, processing of the pre-miR-483 hairpin is biased toward the generation of the 5′ arm. As such, miR-483-5p was found to be elevated in DMD patient serum, whereas miR-483-3p was not (Figure 4). Notably, the classical myomiRs (miR-1, miR-133, and miR-206) were elevated by a greater magnitude than miR-483-5p and were clearly superior in terms of distinguishing between healthy and DMD individuals. However, levels of myomiRs progressively decline with age,^24^, ^28^ likely as a result of a loss of muscle mass and/or the reduced regenerative potential of aged muscle^57^ (similar to the situation with serum creatine kinase [CK]^58^). As such, if serum myomiR levels decline in an exon skipping-treated DMD patient, it is difficult to say whether this represents an improvement or a worsening of pathology. In contrast, miR-483-5p levels were less affected by aging (Figure 4), and so this miRNA offers a potential advantage as a pharmacodynamic biomarker relative to the myomiRs. Importantly, miR-483 is present at much lower absolute level relative to the myomiRs, and so methodological improvements such as pre-amplification after reverse transcription and/or digital PCR may be required for detection and quantification of miR-483-5p in a clinical setting.

Early reports have shown that loss of dystrophin sensitizes muscle fibers to contractile damage,^59^ leading to transient changes in membrane permeability (through physical tears and/or channel activity). The generic muscle damage biomarker CK is widely regarded to leak from dystrophic muscle due to this sarcolemma instability, and similar ideas have been proposed for ex-myomiRs. This assumption seemed reasonable given that the classical myomiRs are among the most abundant sRNA species in skeletal muscle (Figure S7). As such, if ex-miRNAs passively leak from damaged muscle, it might be expected that the most differentially abundant miRNAs in dystrophic serum would be determined simply by those miRNAs that exhibit the highest absolute expression levels in muscle. In the present study, we have directly tested this hypothesis by parallel digital gene expression analyses in both serum and muscle. Although some of the differentially abundant serum miRNAs (e.g., miR-22-3p, miR-133a-3p, miR-133b-3p, miR-206-3p) were indeed among the most highly expressed miRNAs in muscle, a multitude of highly expressed muscle miRNAs were unchanged in *mdx* serum (e.g., miR-126a-3p) (Figure 3). Conversely, lowly abundant muscle miRNAs (e.g., miR-483-3p) are highly elevated in *mdx* serum. These findings are consistent with our previous hypothesis that miRNAs may be selectivity released during the myogenic differentiation that accompanies muscle growth and regeneration, rather than merely as a consequence of passive leakage from damaged muscle.^9^, ^22^, ^28^ Serum myomiR abundance is therefore likely to be a complex function of the regenerative and/or degenerative status of the muscle, overall muscle mass, nuclease stability, and tissue expression levels.^28^ These findings have implications for understanding the clinical relevance of ex-myomiR levels in DMD patient serum.

Other sRNA species (e.g., piRNAs, snRNAs, and rRNAs) were found to be highly abundant in our sequencing libraries (Figures 5 and 6). Many of these ncRNAs were differentially abundant in dystrophic serum, highlighting that these species have potential utility as disease biomarkers. Importantly, the pattern of read density for many of these sRNAs was consistent between serum and muscle, suggesting that these species are processed prior to release or export, as opposed to being random degradation products, and may therefore contribute to normal and dystrophic muscle biology.

piRNAs have previously been observed in mouse muscle, where their functions (if any) are unknown,^46^ and also in other tissues such as the hippocampus^64^ and cerebral cortex.^65^ piRNAs constitute the largest and most diverse class of sRNAs, consisting of 23,439 and 39,986 members in human and mouse, respectively.^66^ Many piRNAs do not show sequence conservation between human and mouse (but are instead syntenically conserved). This lack of conservation may limit the patient relevance of murine studies for the identification of novel biomarkers. Nevertheless, the sequence of piR_000935 (which also overlaps with the Y RNA *Rny1*) is completely conserved between human and mouse.

sRNA-seq is the method of choice for analyzing species such as miRNAs because it is hybridization independent, exhibits low signal to noise, can resolve single-nucleotide differences between related miRNAs (including isomiRs and RNA editing events), and is also capable of discovering new sRNAs given that it is not dependent on an *a priori* list of known genes. However, this methodology is subject to some important limitations. Most notably, sRNA library preparation is known to introduce biases in sequencing data that distort expression analysis.^67^ This is likely due to the adaptor ligation,^68^, ^69^, ^70^ reverse transcription, or PCR steps,^71^ whereby some adaptor pairs or sequence compositions are favored over others. These effects lead to over- or under-representation of specific sRNA reads in the resulting sequenced libraries. Such distortions are also apparent in our data, as exemplified by the massive overrepresentation of miR-1a-3p in both serum and muscle libraries (and consequently a failure to detect elevated miR-1a-3p levels in dystrophic serum), and which may further decrease the dynamic range for detecting other miRNAs. Furthermore, for all three myomiRs, the fold change measured by sRNA-seq was less than that measured by qRT-PCR, suggesting that differences may be underestimated in the sequencing data.^72^ With increased sample size (and therefore statistical power), it might be possible to detect additional differentially expressed miRNAs. Importantly, all key findings in the present study were independently verified using an orthogonal methodology (qRT-PCR) with larger sample sizes to compensate for the technical limitations described above (i.e., reduced dynamic range, sequencing bias, and sub-optimal statistical power, and to rule out false positives as consequence of sequencing artifacts).

The data presented herein constitute the first parallel investigation of the sRNA transcriptome in dystrophic muscle and serum. In particular, the use of sRNA-seq has revealed the complexity of global sRNA expression in ways that are not possible with other methodologies. These analyses provide a wealth of information regarding the differential abundance of miRNAs, and other less well-understood sRNAs, in the context of dystrophin deficiency. In conclusion, this study has identified novel pharmacodynamic biomarkers and offers new insights into the regulation of ex-miRNA release and dystrophic pathophysiology.

## Materials and Methods

### Animal Studies

All animal studies were conducted in accordance to procedures approved by the UK home office (project license 30/2907). Animals used were 14-week-old male wild-type C57BL/10 (C57) and dystrophic C57BL/10ScSn-*Dmd*^*mdx*^/J (*mdx*) mice. Mice were sacrificed by escalating CO_2_ concentration, and blood was collected from the jugular vein and processed as described previously.^73^ Following exsanguination, the diaphragm, gastrocnemius, soleus, and TA were macrodissected and snap-frozen in isopentane pre-chilled on dry ice. 12-week-old male *mdx* mice (n = 4) were injected with a single dose of 12.5 mg/kg Pip6a-PMO conjugate prepared in a sterile saline solution via the tail vein as described previously.^10^, ^74^ Animals were sacrificed 2 weeks post-injection, and serum and tissue were harvested as described above. Pip6a-PMO comprises a PMO moiety (5′-GGCCAAACCTCGGCTTACCTGAAAT-3′) covalently conjugated to an arginine-rich cell-penetrating peptide (Ac-RXRRBRRXRYQFLIRXRBRXRB-OH, where X is aminohexanoyl and B is β-alanine). This compound is designed to induce the specific exclusion of *Dmd* exon 23.

### Human Studies

Serum samples from DMD patients were obtained from Newcastle University through the MRC Centre for Neuromuscular Diseases Biobank. Serum samples from healthy individuals were obtained from Newcastle University (as above) or collected from volunteers at the University of Oxford. All samples were collected according to Biobank standard operating procedures. Collection of serum samples from patients and their use in research have been ethically approved by the NRES Committee North East – Newcastle and North Tyneside 1 in accordance with the Helsinki Declaration. Written informed consent was received from all participants prior to inclusion in the study.

### sRNA Sequencing

RNA was extracted from 200 μL of serum utilizing TRIzol LS and muscle tissues using TRIzol reagent according the manufacturer’s protocols (both Life Technologies, Paisley, UK). RNA concentration was determined using a Qubit Fluorometer (Life Technologies, Paisley, UK). For tissue samples, RNA integrity was determined using a High Sensitivity D1000 ScreenTape station (Agilent Technologies, Lanarkshire, UK). sRNA libraries were generated using the NEBNext Multiplex sRNA Library Prep Set for Illumina kits (sets 1 [E7300S] and 2 [E7580S]; NEB, Ipswich, MA, USA) according to manufacturer’s instructions with minor alterations. In brief, 480 ng of RNA for muscle and 10 ng for serum were used as input material for adaptor ligation and cDNA synthesis. For the serum samples, 3′ SR adaptor, 3′ SR RT primer, and 5′ SR adaptor were diluted 1:3. Subsequently, the libraries were amplified for 15 and 20 cycles for muscle and serum samples, respectively. Subsequently, the barcoded samples were size selected on a 6% Novex Tris-Borate-EDTA (TBE) PAGE gel (Life Technologies, Paisley, UK), purified with the NucleoSpin Gel and PCR Clean-up kit (Macherey-Nagel, Düren, Germany), and quantified using the KAPA Library Quantification Kit (Kapa Biosystems, London, UK). Samples were pooled at equimolar ratio into two multiplex libraries containing 24 samples each. Next, the libraries were pre-amplified for four cycles and the appropriate size of purified fragments confirmed on the ScreenTape station. Clustering was performed on the Illumina cBot instrument, and samples were single-end sequenced on HiSeq 2500 (HiSeq Control Software 2.2.58/RTA 1.18.64) with a 1 × 51 setup using the HiSeq Rapid SBS Kit v2 (all Illumina, San Diego, CA, USA). All sRNA-seq data are deposited in NCBI SRA: SRP102619. A complete description of the sRNA-seq bioinformatics analysis is described in detail in the Supplemental Materials and Methods. The DESeq (v1.26.0)^43^ R package was used to determine differentially expressed ncRNAs (negative binomial distribution test, unpaired analysis). DESeq was used to calculate size factors and raw read counts scaled accordingly to normalize for library size. Lowly abundant sRNA species were filtered out if the sum of counts across all samples was less than 100. Reported p values were corrected for multiple comparisons using the Benjamini-Hochberg method. Adjusted p values < 0.05 were considered statistically significant. Differential expression was calculated between the *mdx* and C57 groups only, although DESeq was used to calculate scale factors and normalized counts for all groups (including PPMO treated). The PPMO group was utilized to quantitatively assess the response of the miRNome to exon skipping therapy, and key findings were validated by qRT-PCR (with increased sample size and statistical power).

### sRNA qRT-PCR

We have previously described in detail our methods for the detection and quantification of ex-miRNAs in murine biofluids.^73^, ^75^ All qRT-PCR studies were designed to comply with the Minimum Information for Publication of Quantitative Real-Time PCR Experiments (MIQE) guidelines where possible. To monitor variation in extraction efficiencies for biofluid samples, we added 3 μL of a 5 nM synthetic miRNA oligonucleotide, cel-miR-39 (5′-UCACCGGGUGUAAAUCAGCUUG-3′) (IDT, Leuven, Belgium), to each sample at the phenol extraction stage. cDNA synthesis was performed using the MicroRNA Reverse Transcription Kit (Life Technologies) and appropriate miRNA-specific hairpin RT primer according to the manufacturer’s instructions (assay IDs are listed in Tables S2 and S3). Subsequently, miRNAs were amplified using sRNA TaqMan assays and TaqMan Gene Expression Master Mix on a Step-One Real-Time PCR instrument (all Life Technologies). For absolute quantification, sample miRNA quantities were compared with a 10-fold dilution series of synthetic miRNA oligonucleotides (IDT) spiked in at the RT stage. This technique enables the comparison of measurements between experiments and also allows for direct comparison between different miRNA assays. When appropriate, relative quantification was performed using the Pfaffl method.^76^ Sample quantities were normalized to cel-miR-39 levels in the case of biofluids and to miR-16-5p levels for tissue samples. To obtain serum miRNA concentrations (copy numbers per milliliter), the ratio of input volume used for extraction and RNA resuspension volume was calculated, and measured miRNA copy numbers scaled accordingly.

### Statistics

Heatmap and clustering analyses were performed in MeV (Multiple experiment Viewer) (The Institute for Genomic Research, Rockville, MD, USA).^77^ Principal component analysis (PCA) was performed in R using the *prcomp* function. Plots were produced using GraphPad Prism 5 (GraphPad Software, La Jolla, CA, USA). The following additional analyses were performed in GraphPad Prism 5: unpaired t test, one-way ANOVA, Bonferroni *post hoc* test, Pearson and Spearman correlation analyses, and ROC curve analysis. Venn diagrams were produced with Venny v2.1.0 (http://bioinfogp.cnb.csic.es/tools/venny). Statistical analysis of sRNA-seq data is described in detail in the Supplemental Materials and Methods.

## Author Contributions

Conceptualization, T.C.R. and M.J.A.W.; Investigation, A.M.L.C.-S., H.S., K.K., A.B., C.G., and T.C.R.; Formal Analysis, A.M.L.C.-S., S.G., J.R.H., and T.C.R.; Resources, L.O., A.R., and H.L.; Writing – Original Draft, T.C.R. and A.M.L.C.-S.; Writing – Review & Editing, all authors; Supervision, T.C.R., J.O.W., M.J.A.W., P.L.P., and S.E.A.; Funding Acquisition, M.J.A.W. and S.E.A.

## Conflicts of Interest

C.G. and M.J.A.W. are founders of Pepgen Ltd., which aims to commercialize peptide technology similar to that utilized in this manuscript. The remaining authors declare no competing interests.

## Supplemental Information

Document S1. Supplemental Materials and Methods, Figures S1–S19, and Tables S1–S3

Data S1. miRNA Master Data File

Data S2. Human miR-483 Target Predictions

Data S3. Target Predictions for Novel miRNAs

Data S4. ncRNA Master Data File

Document S2. Article plus Supplemental Information
