# Supplementary material for: Comprehensive RNA-Sequencing Analysis in Serum and Muscle Reveals Novel Small RNA Signatures with Biomarker Potential for DMD
Source: Mol Ther Nucleic Acids. 2018 Aug 17;13:1–15. doi: 10.1016/j.omtn.2018.08.005 (PMC6140421; doi:10.1016/j.omtn.2018.08.005)
Supplement: Document S1. Supplemental Materials and Methods, Figures S1–S19, and Tables S1–S3 [file mmc1.pdf]

OMTN, Volume *13*

Supplemental Information

Comprehensive RNA-Sequencing Analysis in Serum and Muscle Reveals Novel Small RNA Signatures with Biomarker Potential for DMD

Anna M.L. Coenen-Stass, Helena Sork, Sole Gatto, Caroline Godfrey, Amarjit Bhomra, Kaarel Krjutškov, Jonathan R. Hart, Jakub O. Westholm, Liz O'Donovan, Andreas Roos, Hanns Lochmüller, Pier Lorenzo Puri, Samir EL Andaloussi, Matthew J.A. Wood, and Thomas C. Roberts

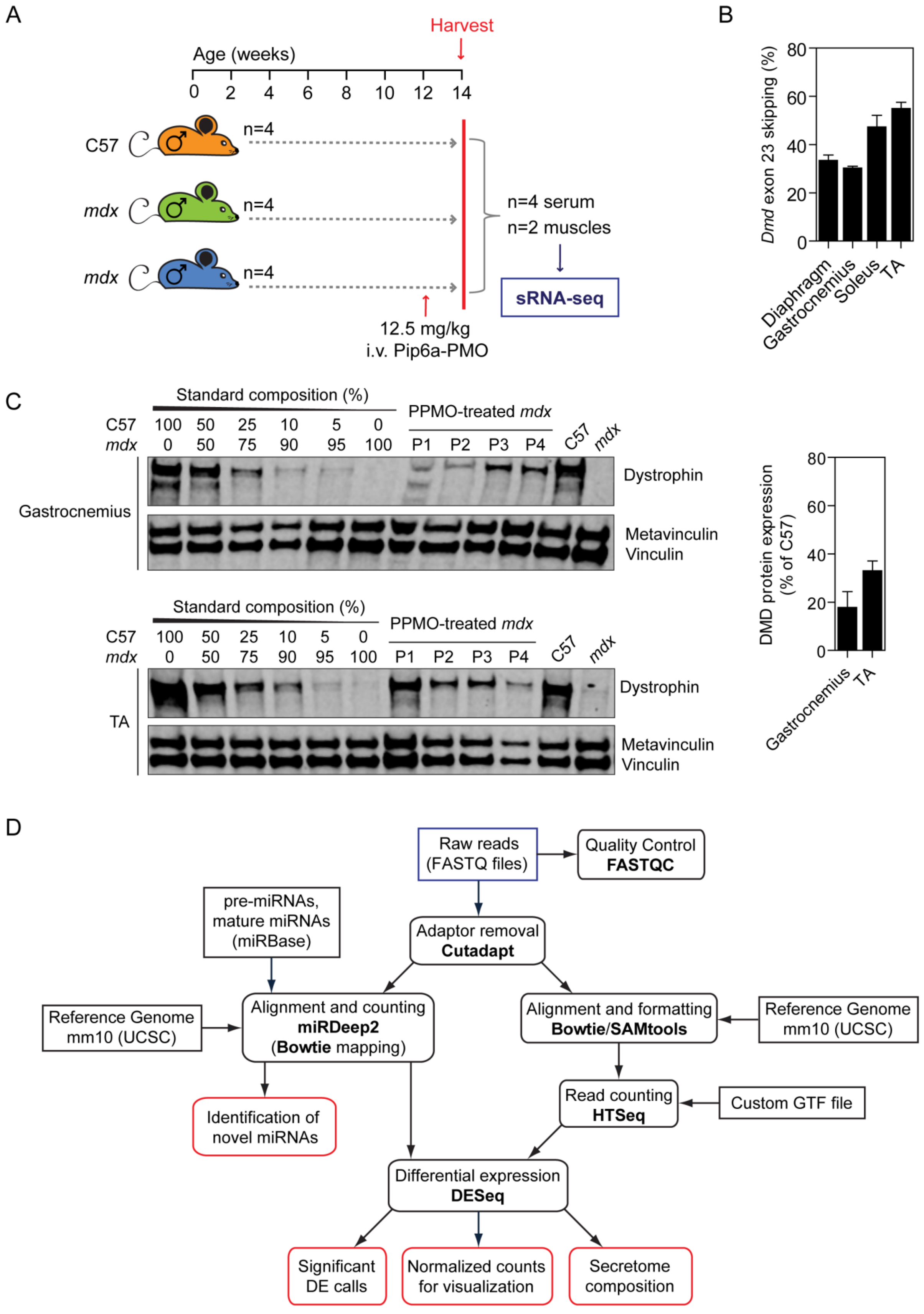
A
Harvest
Age (weeks)
0 2 4 6 8 10 12 14
C57
mdx
mdx
n=4
n=4
n=4
n=4 serum
n=2 muscles
sRNA-seq
12.5 mg/kg
i.v. Pip6a-PMO
B
Dmd exon 23 skipping (%)
0 20 40 60 80
Diaphragm
Gastrocnemius
Soleus
TA
C
Standard composition (%)
PPMO-treated mdx
C57 100 50 25 10 5 0
mdx 0 50 75 90 95 100
P1 P2 P3 P4 C57 mdx
Gastrocnemius
Dystrophin
Metavinculin
Vinculin
TA
DMD protein expression (% of C57)
0 20 40 60 80
Gastrocnemius
TA
D
Raw reads (FASTQ files)
Quality Control FASTQC
pre-miRNAs, mature miRNAs (miRBase)
Adaptor removal Cutadapt
Reference Genome mm10 (UCSC)
Alignment and counting miRDeep2 (Bowtie mapping)
Alignment and formatting Bowtie/SAMtools
Reference Genome mm10 (UCSC)
Read counting HTSeq
Custom GTF file
Identification of novel miRNAs
Differential expression DESeq
Significant DE calls
Normalized counts for visualization
Secretome composition

Figure S1

Experimental design and sequencing analysis pipeline

(A) Experimental design of the small RNA sequencing study. Libraries were prepared utilizing RNA extracted from serum and four distinct muscles (Diaphragm, Gastrocnemius, Soleus and TA) of 14 week old *mdx*, PPMO-treated *mdx* and wild-type (C57) mice. *Dmd* exon-23 skipping in PPMO-treated animals was confirmed by (B) qRT-PCR (n = 3) and (C) Western blot (n = 4). Values are mean+SEM. (D) Schematic of sequence analysis pipeline.

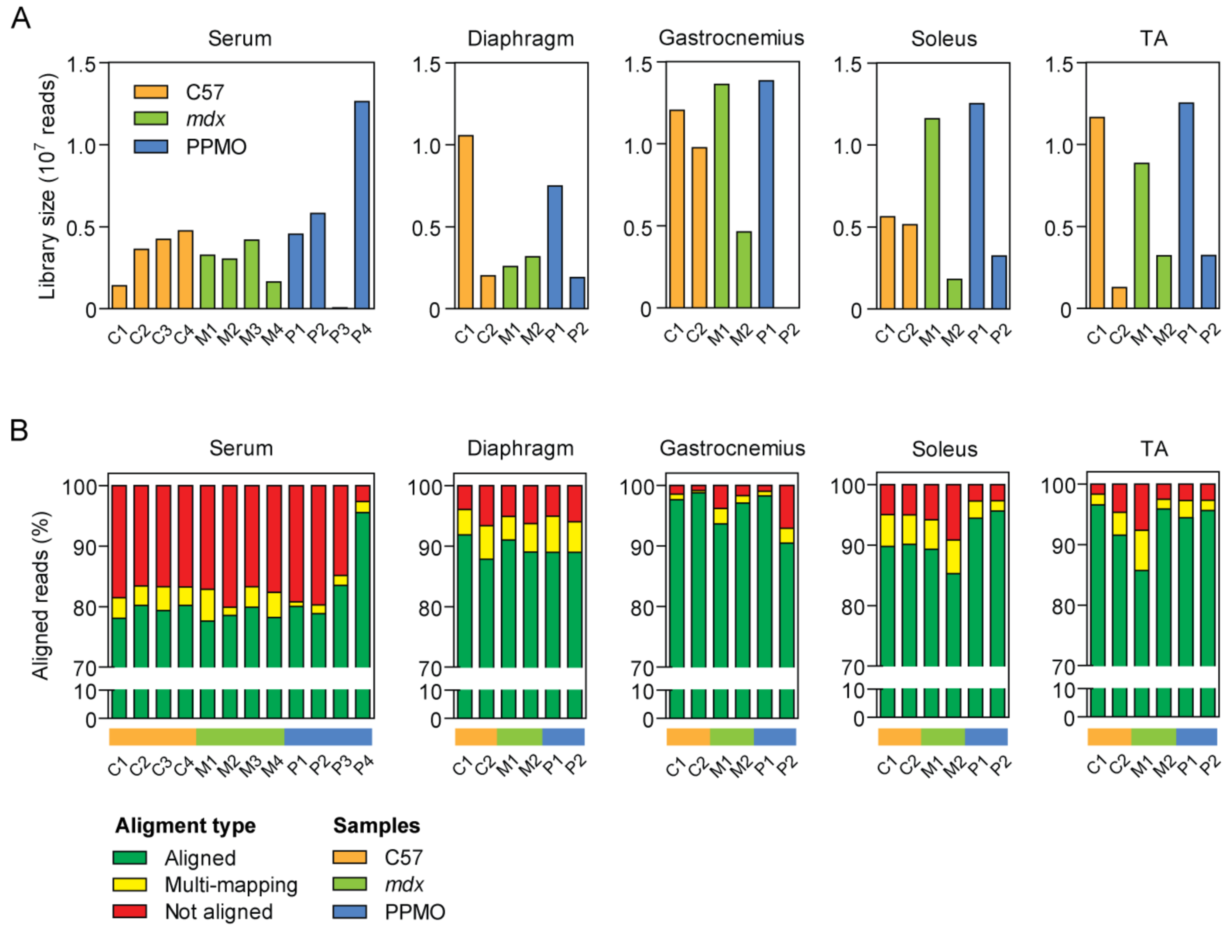

Figure S2

Library sizes and Bowtie mapping statistics

(A) Library sizes for all serum and muscle samples. Libraries were generally of consistent sizes with the exception of two atypical serum samples P3 and P4, and the gastrocnemius sample P2. (B) Mapping statistics after Bowtie alignment of all sample libraries to the mouse genome (mm10). Reads were classified as aligned, not aligned, or multi-mapping. Reads mapping to the mouse genome more than 20 times were scored as 'Not-aligned' using the –m20 argument in Bowtie. Quality of mapping was generally high and consistent between samples. Notably, fewer reads were mapped in the serum libraries than in the muscle samples.

A

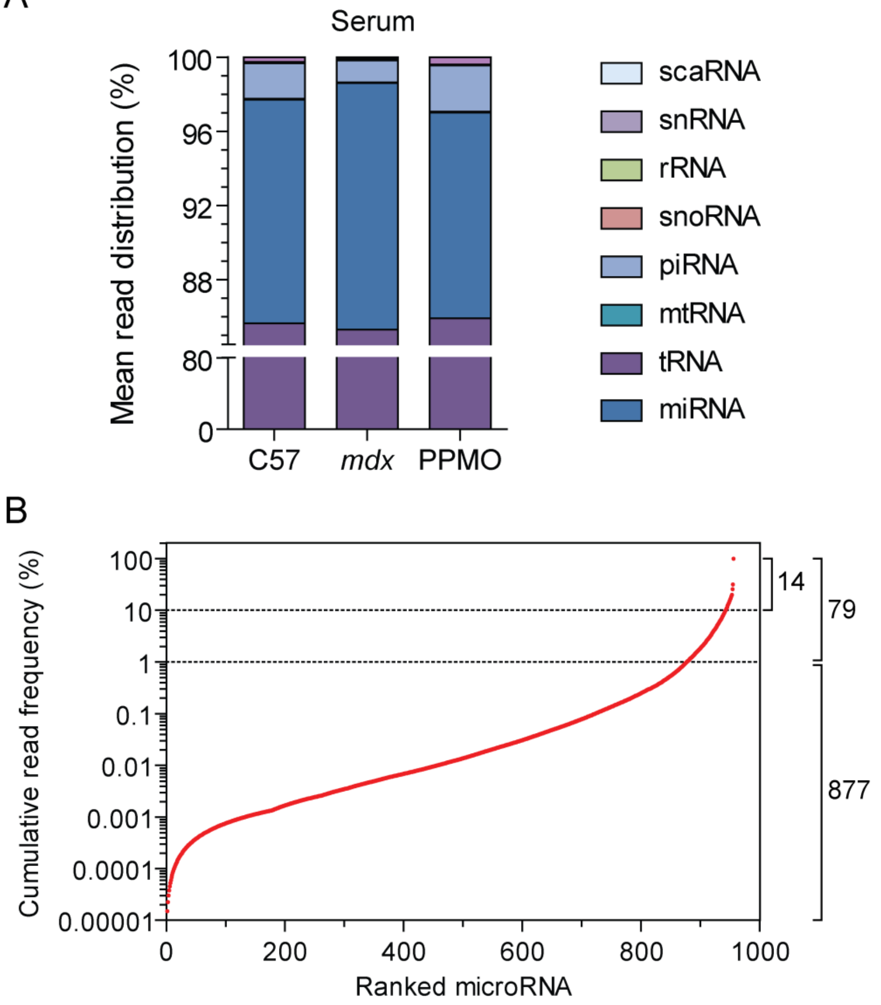

C

	Counts
**miR-1a-3p**	9118512
**miR-486a-5p**	773125
**miR-486b-5p**	773125
**miR-378a-3p**	190896
**miR-133a-3p**	186395
**miR-143-3p**	179102
**miR-148a-3p**	136692
**miR-92a-3p**	128577
**miR-21a-5p**	105527
**miR-22-3p**	102199
**miR-30a-5p**	98768
**miR-206-3p**	93207
**miR-26a-5p**	83504
**miR-99a-5p**	74528

Figure S3

Estimation of miRNA proportions and dynamic range in serum

(A) Distribution of mapped reads to ncRNA categories comparing experimental groups in serum. (B) miRNA counts data for the serum libraries were pooled and miRNAs ranked by abundance. The percentage of the total miRNA signal was estimated for each individual miRNA by dividing the number of counts per miRNA by the total number of counts. The resulting data are shown in a cumulative frequency plot. 14 miRNAs make up 90% of the total miRNA reads, with 68.4% of all miRNA reads mapping to miR-1a-3p. The top 79 miRNAs comprised 99% of the total reads, with the remaining 877 least abundant miRNAs contributing to 1% of the total miRNA signal. (C) The identities of the 14 most abundant serum miRNAs and their total counts data are listed.

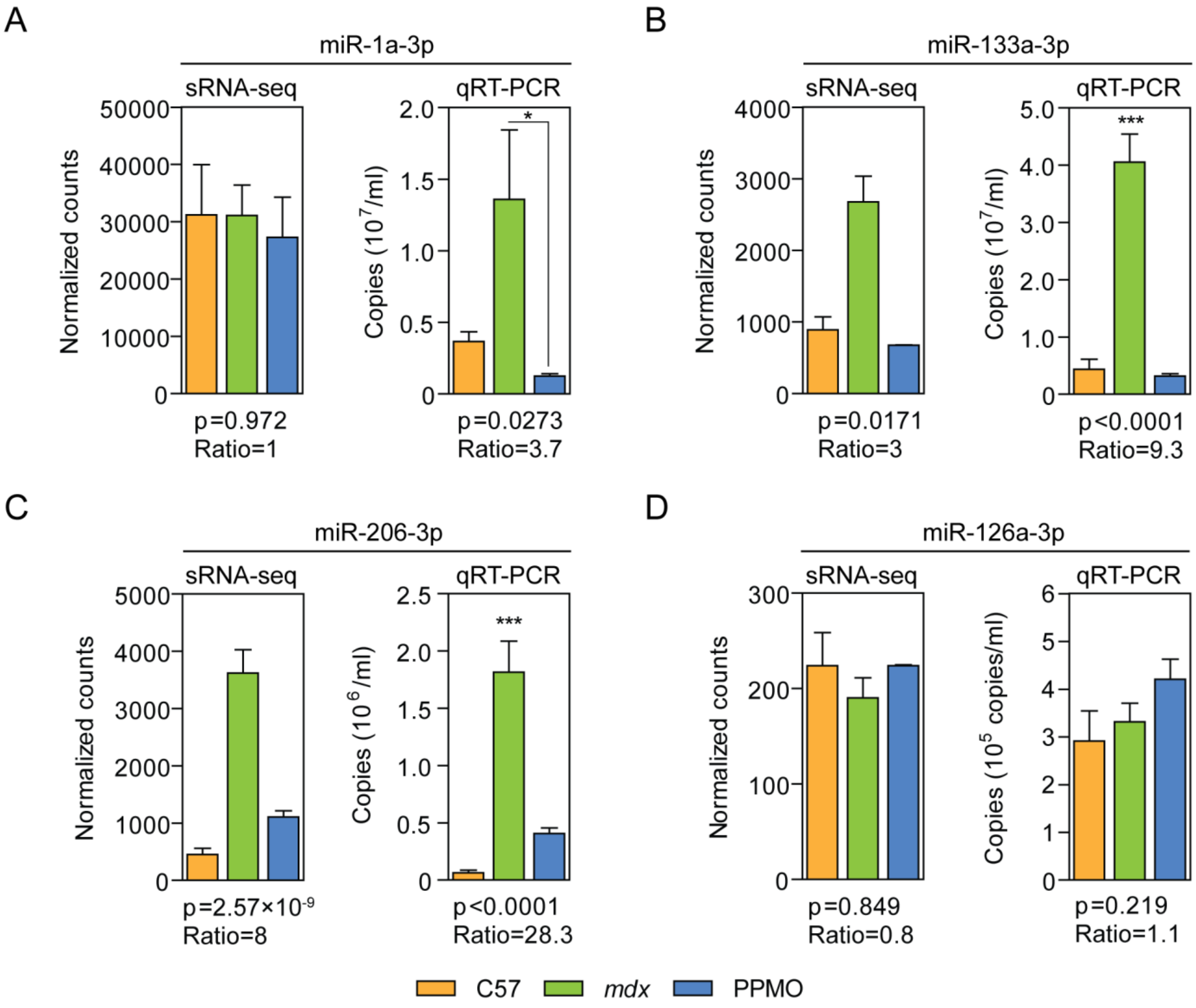

Figure S4

Validation of serum myomiR abundance data

Serum abundance data for the myomiRs (A) miR-1a-3p, (B) miR-133a-3p, and (C) miR-206-3p are shown as determined by sRNA-seq and sRNA TaqMan qRT-PCR. (D) Data for the abundant non-myomiR control miRNA, miR-126a-3p, that was not expected to change are shown for comparison. All values are mean+SEM, n = 4. p-values represent negative binomial distribution test (with Benjamini-Hochberg correction for multiple comparisons) or one-way ANOVA for sRNA-seq and qRT-PCR respectively. *mdx* vs C57 fold change are indicated, *$p < 0.05$, ***$p < 0.001$, Bonferroni *post hoc* test. Statistical comparisons are to the C57 control group unless otherwise indicated.

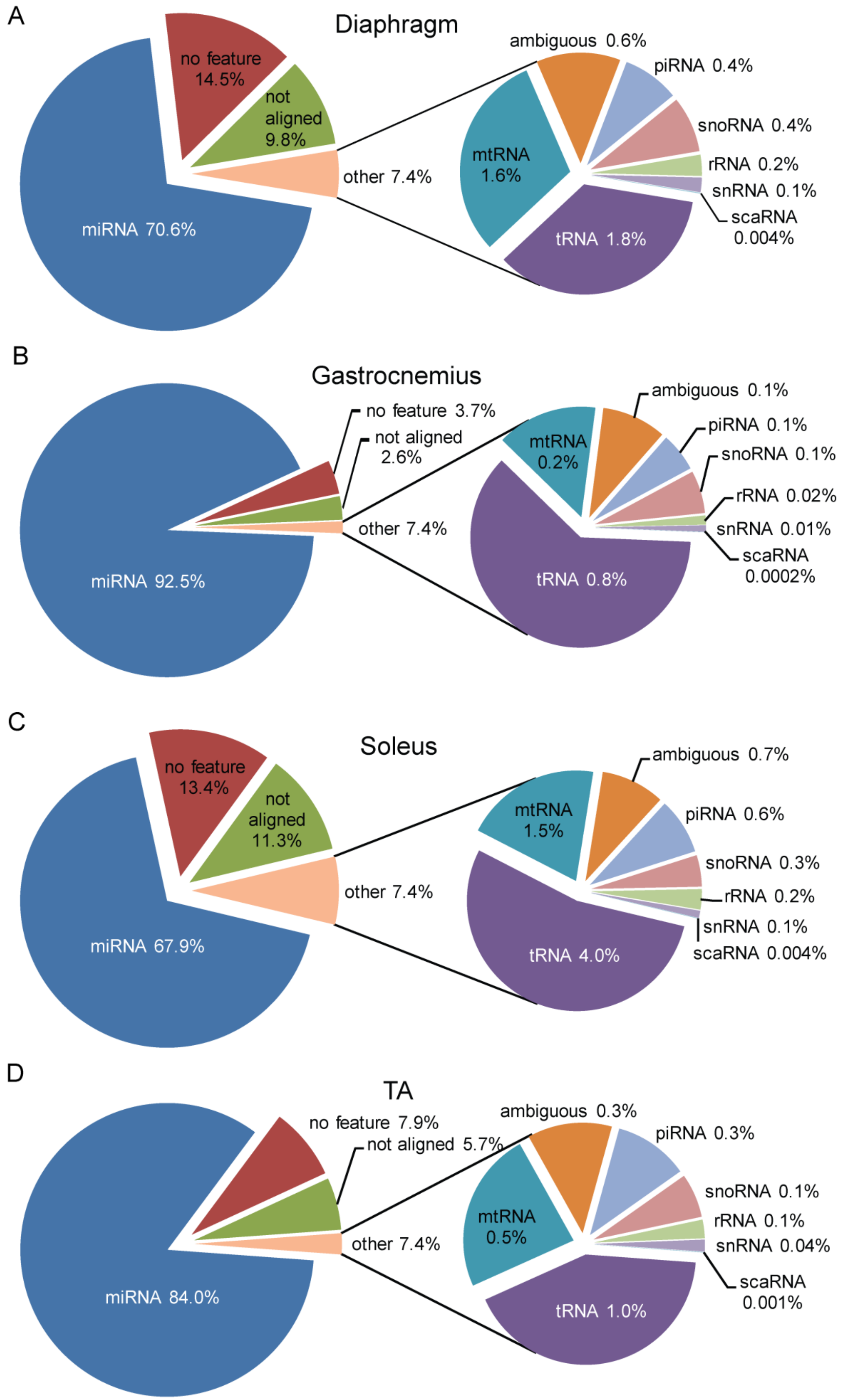
A
Diaphragm
no feature 14.5%
not aligned 9.8%
other 7.4%
miRNA 70.6%
ambiguous 0.6%
piRNA 0.4%
snoRNA 0.4%
rRNA 0.2%
snRNA 0.1%
scaRNA 0.004%
mtRNA 1.6%
tRNA 1.8%
B
Gastrocnemius
no feature 3.7%
not aligned 2.6%
other 7.4%
miRNA 92.5%
ambiguous 0.1%
piRNA 0.1%
snoRNA 0.1%
rRNA 0.02%
snRNA 0.01%
scaRNA 0.0002%
mtRNA 0.2%
tRNA 0.8%
C
Soleus
no feature 13.4%
not aligned 11.3%
other 7.4%
miRNA 67.9%
ambiguous 0.7%
piRNA 0.6%
snoRNA 0.3%
rRNA 0.2%
snRNA 0.1%
scaRNA 0.004%
mtRNA 1.5%
tRNA 4.0%
D
TA
no feature 7.9%
not aligned 5.7%
other 7.4%
miRNA 84.0%
ambiguous 0.3%
piRNA 0.3%
snoRNA 0.1%
rRNA 0.1%
snRNA 0.04%
scaRNA 0.001%
mtRNA 0.5%
tRNA 1.0%

Figure S5

sRNA library composition for muscle samples

Mapped sRNA reads from muscle libraries were sorted into the following ncRNA classes; miRNA, tRNA, rRNA, snRNA, snoRA, scaRNA, mtRNA, and piRNA. Pie charts show the percentage of reads mapping to each of the ncRNA categories for (A) diaphragm, (B) gastrocnemius, (C) soleus, and (D) TA.

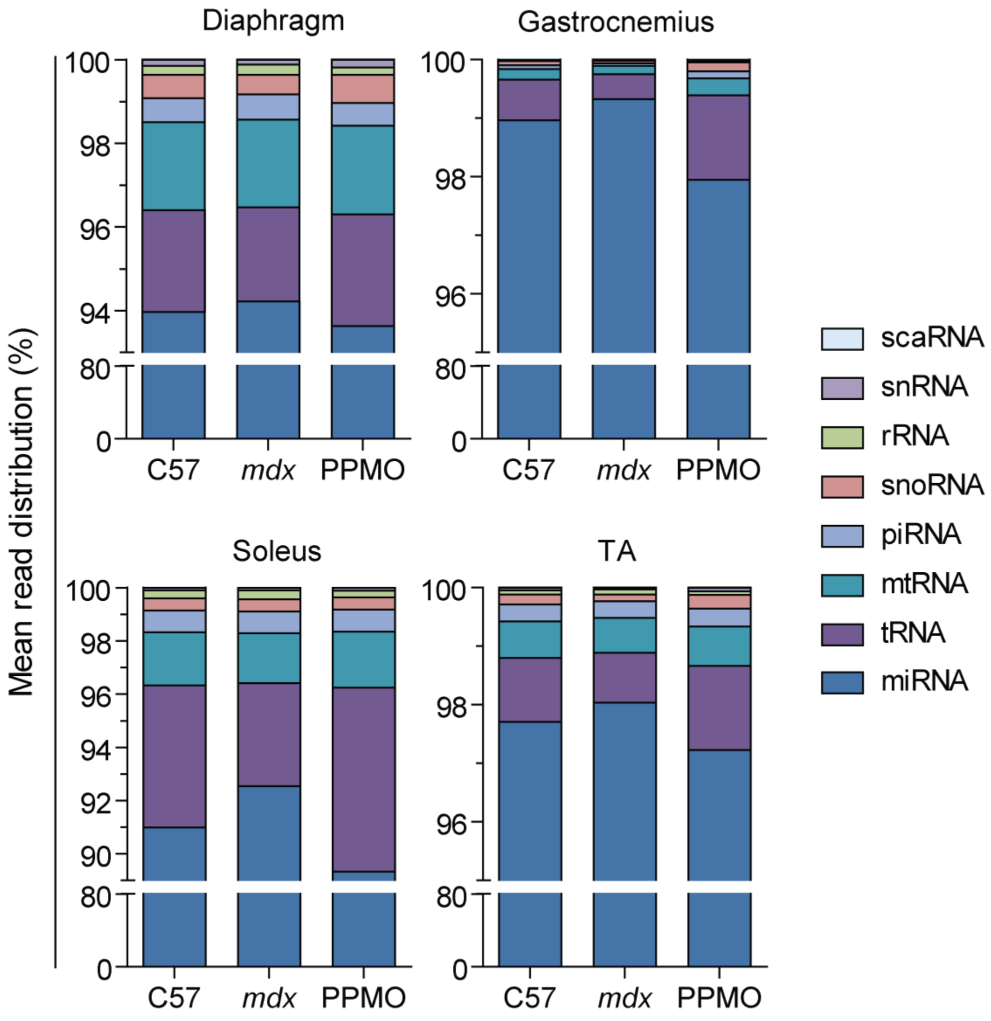

Figure S6
Distribution of mapped reads to ncRNA categories comparing experimental groups in each muscle type

A

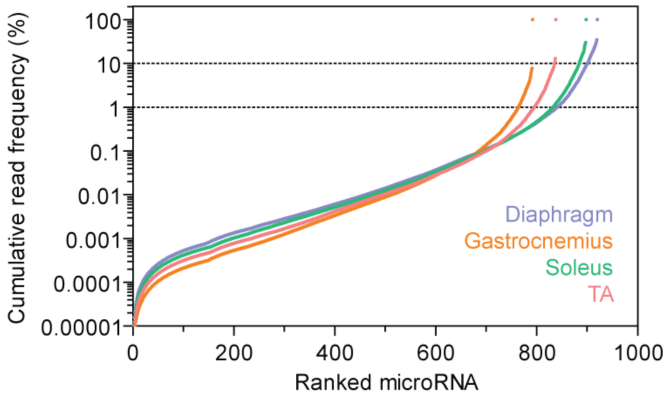

B

	Diaphragm	Gastrocnemius	Soleus	TA
**Top 10%**	20	1	14	3
**Top 99%**	80	29	66	43
**Bottom 1%**	840	763	832	795
**Total**	920	792	898	838

C

	Diaphragm
**miR-1a-3p**	12511403
**miR-143-3p**	737353
**miR-378a-3p**	530000
**miR-133a-3p**	431930
**miR-21a-5p**	310626
**miR-30a-5p**	280418
**miR-26a-5p**	237353
**miR-126a-3p**	231202
**let-7f-5p**	208433
**miR-22-3p**	203280
**miR-99a-5p**	195357
**let-7i-5p**	191705
**miR-27b-3p**	181881
**miR-206-3p**	181119
**let-7g-5p**	180038
**miR-30d-5p**	155938
**miR-148a-3p**	140666
**miR-378c**	103136
**miR-10a-5p**	97156
**miR-133b-3p**	93694

	Gastrocnemius
**miR-1a-3p**	43849055
**miR-378a-3p**	407116
**miR-133a-3p**	370982
**miR-143-3p**	362159
**miR-206-3p**	318402
**miR-21a-5p**	270077
**miR-30a-5p**	165349
**miR-26a-5p**	120594
**miR-22-3p**	108205
**let-7f-5p**	98602
**let-7i-5p**	98581
**miR-99a-5p**	85059
**miR-30d-5p**	78875
**let-7g-5p**	69854
**miR-133b-3p**	69439
**miR-27b-3p**	69198
**miR-126a-3p**	60751
**miR-378c**	54209
**miR-486a-5p**	52721
**miR-486b-5p**	52721

	Soleus
**miR-1a-3p**	16814571
**miR-143-3p**	776128
**miR-133a-3p**	696647
**miR-206-3p**	672618
**miR-378a-3p**	557479
**miR-22-3p**	296763
**miR-30a-5p**	273760
**miR-27b-3p**	263580
**miR-26a-5p**	250803
**miR-126a-3p**	235832
**miR-21a-5p**	234601
**miR-99a-5p**	197106
**miR-30d-5p**	188951
**let-7f-5p**	178703
**let-7i-5p**	172971
**let-7g-5p**	152090
**miR-133b-3p**	138205
**miR-378c**	118929
**miR-24-3p**	93440
**miR-100-5p**	83577

	TA
**miR-1a-3p**	27823615
**miR-133a-3p**	568379
**miR-378a-3p**	440393
**miR-143-3p**	427295
**miR-30a-5p**	193628
**miR-21a-5p**	165828
**miR-22-3p**	157734
**miR-26a-5p**	145234
**miR-206-3p**	120583
**miR-126a-3p**	116946
**let-7i-5p**	103331
**miR-133b-3p**	102757
**miR-30d-5p**	99393
**miR-27b-3p**	95075
**let-7f-5p**	94284
**miR-99a-5p**	91926
**let-7g-5p**	82700
**miR-378c**	76125
**miR-100-5p**	68058
**miR-486a-5p**	62549

D

Common miRNAs in top 20 most abundant lists

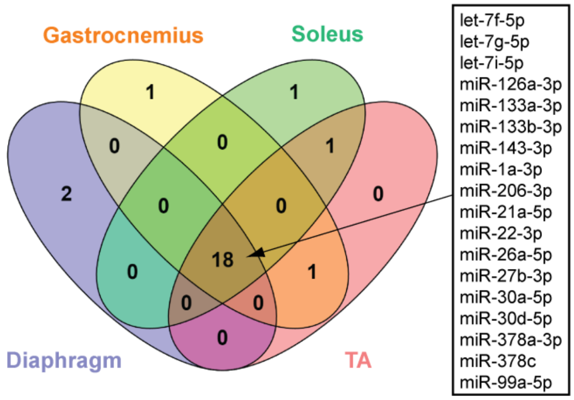

Figure S7

Estimation of miRNA proportions and dynamic range in muscle

miRNA counts data for the muscle libraries were pooled and miRNAs ranked by abundance. The percentage of the total miRNA signal was estimated for each individual miRNA by dividing the number of counts/miRNA by the total number of miRNA counts. The resulting data are shown in the cumulative frequency plot (A). The number of miRNAs that comprise the top 10%, the top 99%, and the bottom 1% of all miRNA reads for each muscle are listed in the table (B). (C) The top 20 highest abundant miRNAs are shown for each muscle, and the overlap between these lists indicated in the Venn diagram (D).

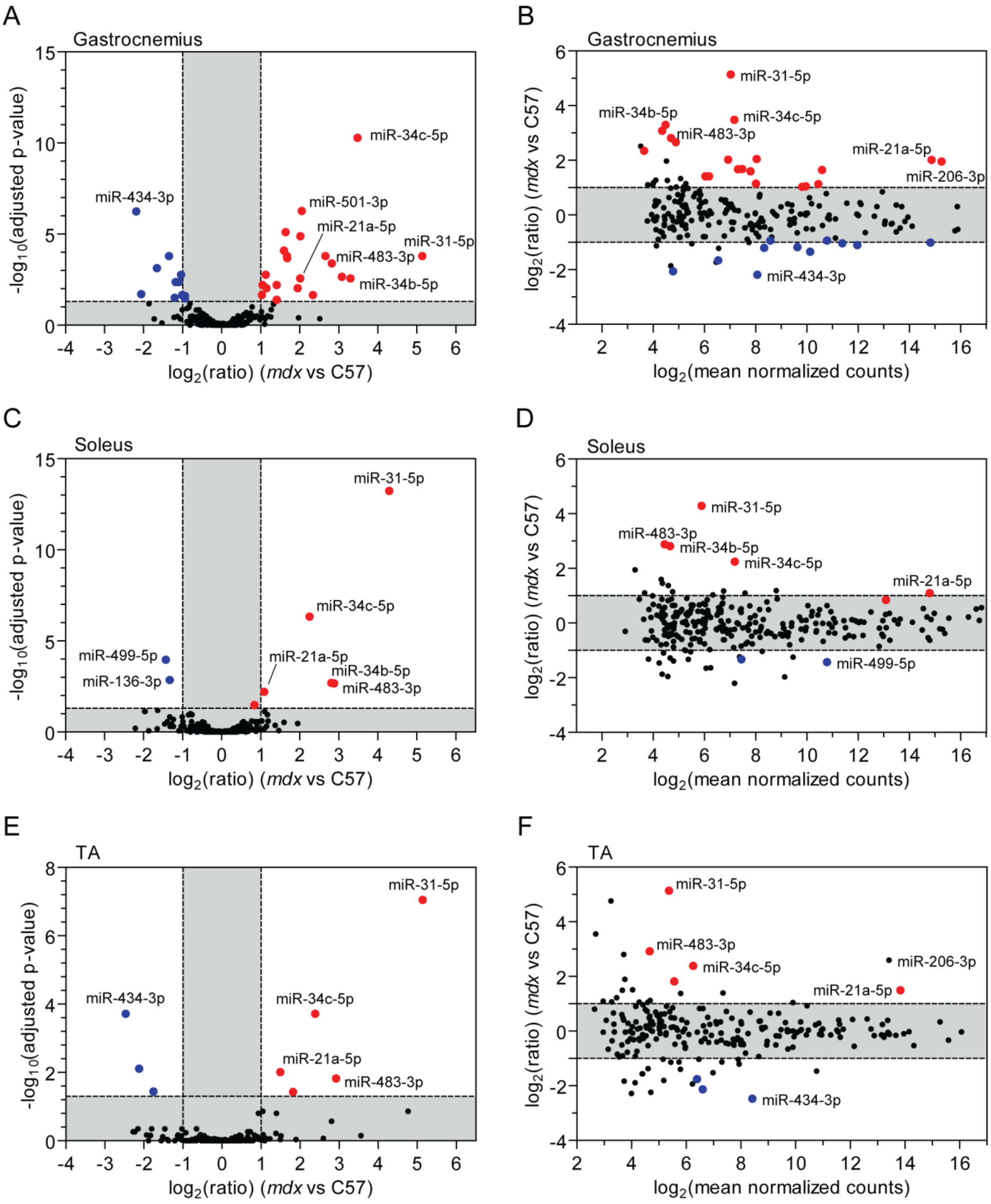

Figure S8

Differential miRNA expression in *mdx* gastrocnemius, soleus and TA muscles

Differential expression of miRNAs in *mdx* muscle relative to wild-type controls as visualized by volcano and MA plots for (A,B) gastrocnemius, (C,D) soleus, and (E,F) TA. Statistically significant changes are highlighted in red and blue (for elevated and reduced levels in *mdx* serum respectively). Labels are shown for miRNAs of interest.

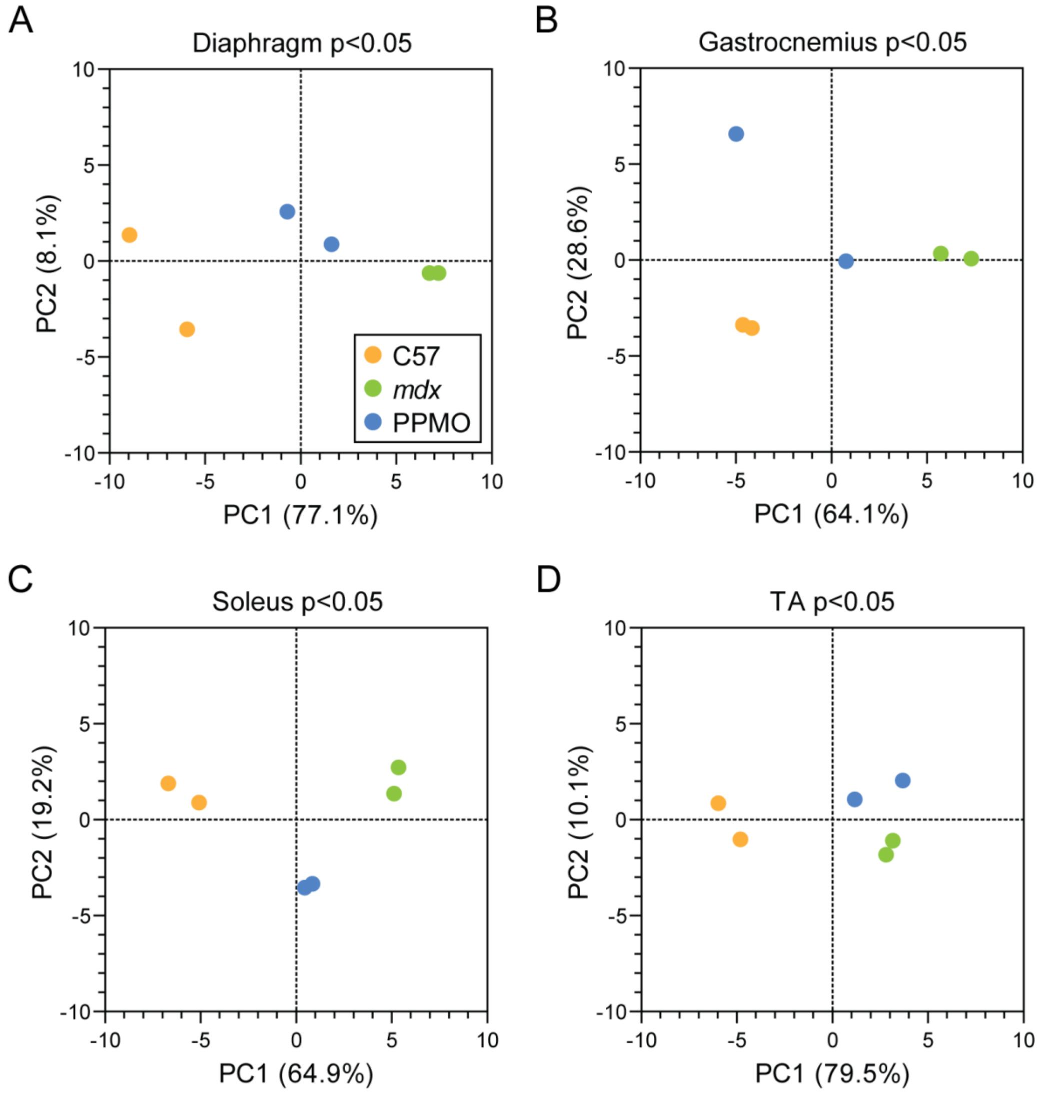

Figure S9
Principal component analysis of miRNA expression after PPMO treatment in muscle
Principal component analysis of statistically significant ($p < 0.05$) miRNA expression ratios comparing wild-type (C57), dystrophic (*mdx*), and PPMO-treated *mdx* mice in (A) diaphragm, (B) gastrocnemius, (C) soleus, and (D) TA muscles.

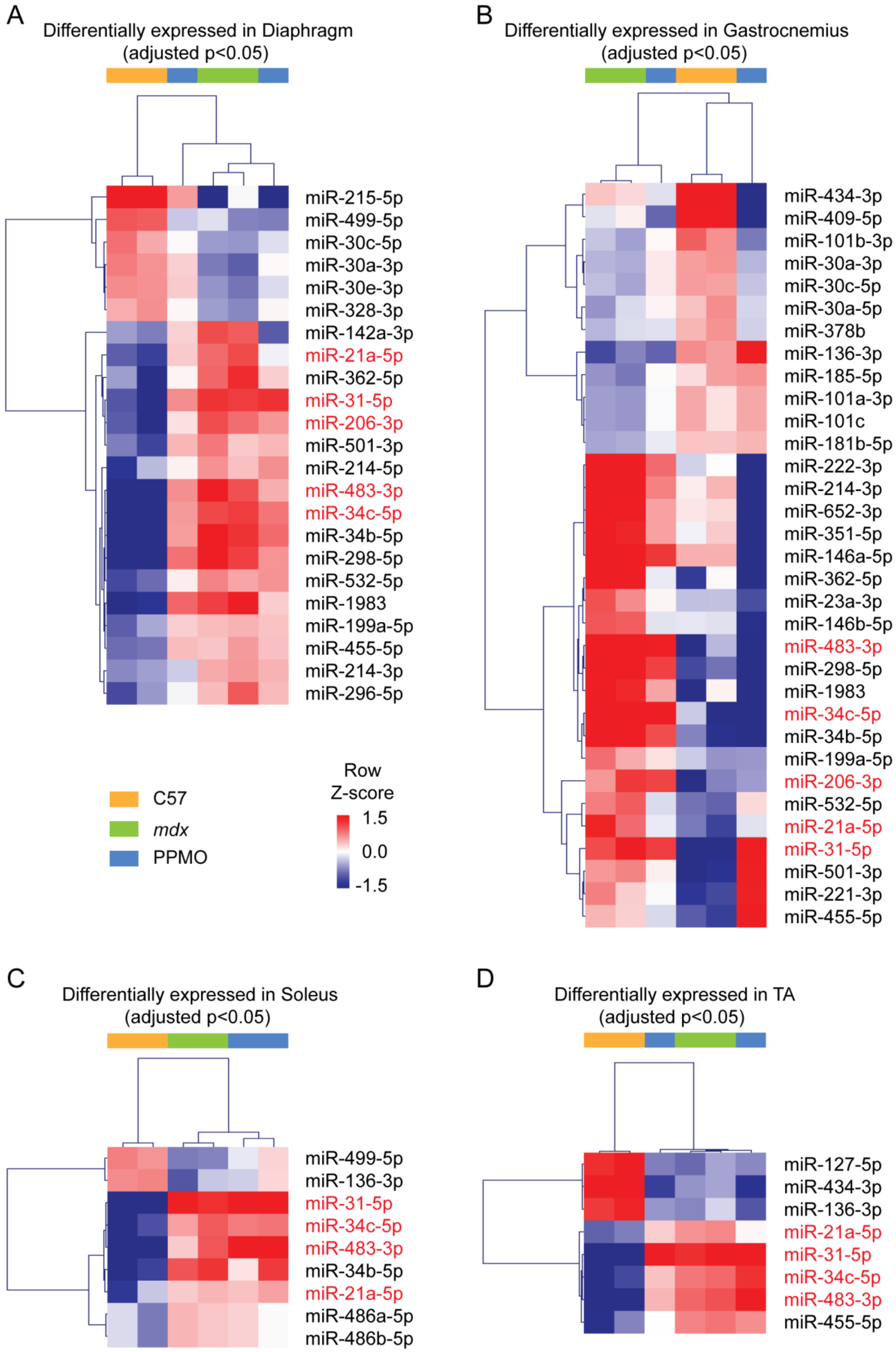
A
Differentially expressed in Diaphragm
(adjusted p<0.05)
miR-215-5p
miR-499-5p
miR-30c-5p
miR-30a-3p
miR-30e-3p
miR-328-3p
miR-142a-3p
miR-21a-5p
miR-362-5p
miR-31-5p
miR-206-3p
miR-501-3p
miR-214-5p
miR-483-3p
miR-34c-5p
miR-34b-5p
miR-298-5p
miR-532-5p
miR-1983
miR-199a-5p
miR-455-5p
miR-214-3p
miR-296-5p
C57
mdx
PPMO
Row
Z-score
1.5
0.0
-1.5
B
Differentially expressed in Gastrocnemius
(adjusted p<0.05)
miR-434-3p
miR-409-5p
miR-101b-3p
miR-30a-3p
miR-30c-5p
miR-30a-5p
miR-378b
miR-136-3p
miR-185-5p
miR-101a-3p
miR-101c
miR-181b-5p
miR-222-3p
miR-214-3p
miR-652-3p
miR-351-5p
miR-146a-5p
miR-362-5p
miR-23a-3p
miR-146b-5p
miR-483-3p
miR-298-5p
miR-1983
miR-34c-5p
miR-34b-5p
miR-199a-5p
miR-206-3p
miR-532-5p
miR-21a-5p
miR-31-5p
miR-501-3p
miR-221-3p
miR-455-5p
C
Differentially expressed in Soleus
(adjusted p<0.05)
miR-499-5p
miR-136-3p
miR-31-5p
miR-34c-5p
miR-483-3p
miR-34b-5p
miR-21a-5p
miR-486a-5p
miR-486b-5p
D
Differentially expressed in TA
(adjusted p<0.05)
miR-127-5p
miR-434-3p
miR-136-3p
miR-21a-5p
miR-31-5p
miR-34c-5p
miR-483-3p
miR-455-5p

Figure S10

Heatmap analysis of miRNA expression after PPMO treatment in muscle

Statistically significant (adjusted $p < 0.05$) miRNA expression ratios were visualized by hierarchical clustering analysis and heatmaps generated comparing wild-type (C57), dystrophic (*mdx*), and PPMO-treated *mdx* mice in (A) diaphragm, (B) gastrocnemius, (C) soleus, and (D) TA muscles. Scale bars show mean-centered, log_2 normalized counts (row Z-score) where red and blue indicate higher and lower than mean expression respectively. Names of miRNAs of particular interest are highlighted in red.

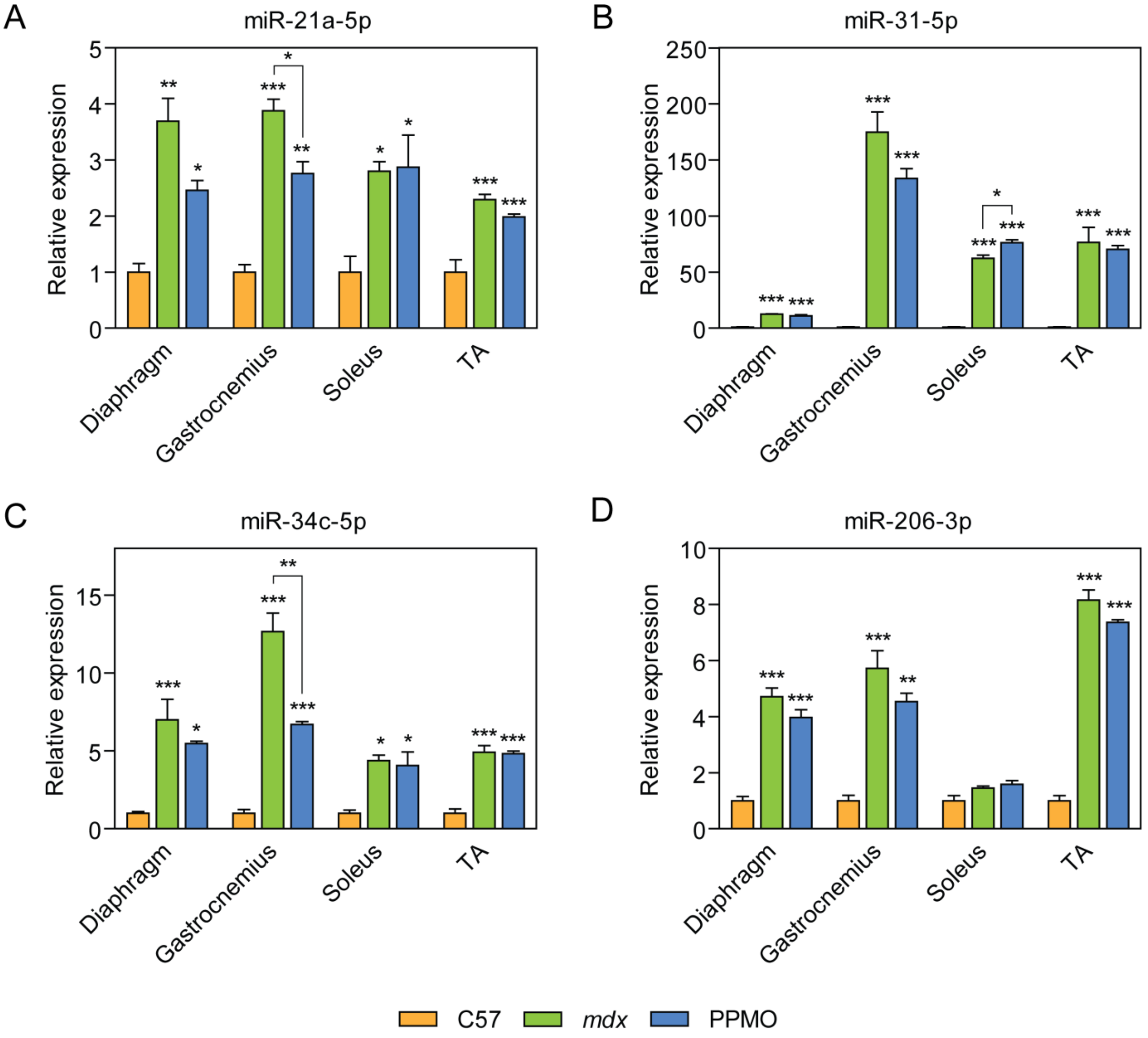

Figure S11

qRT-PCR validation of muscle miRNA expression

Expression of four key miRNAs (miR-21a-5p, miR-31-5p, miR-34c-5p, and miR-206-3p) was measured by qRT-PCR for C57, *mdx* and PPMO-treated samples in (A) Diaphragm, (B) Gastrocnemius, (C) Soleus and (D) TA. miRNA levels were normalized to miR-16-5p expression and all expression ratios scaled such that the mean of the C57 group was returned to one for each muscle. Values are mean+SEM, n = 3, *$p < 0.05$, **$p < 0.01$, ***$p < 0.001$, one-way ANOVA with Bonferroni *post hoc* test. All statistical comparisons indicated are relative to the C57 control group unless otherwise indicated.

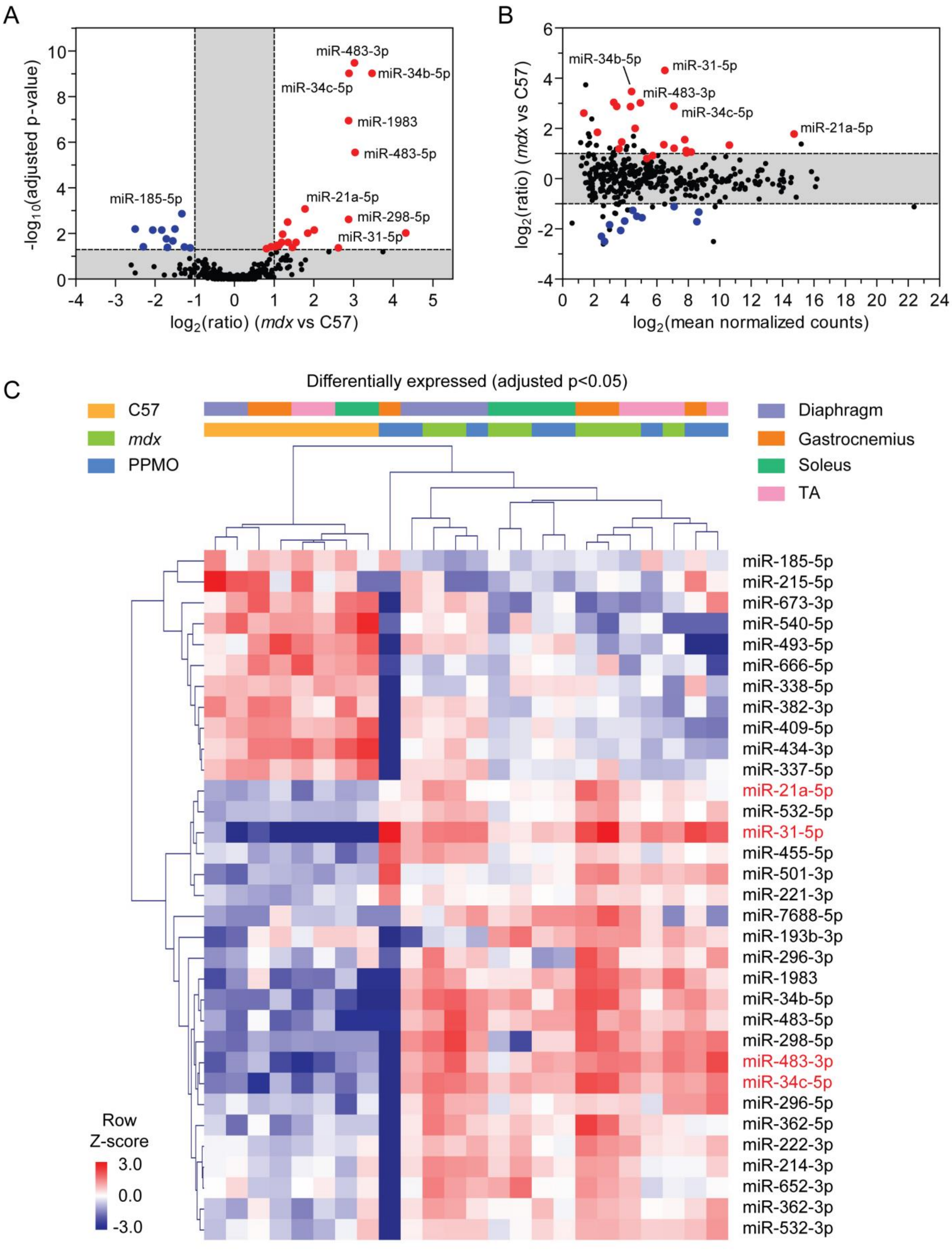

A
B
C
-log10(adjusted p-value)
log2(ratio) (mdx vs C57)
miR-483-3p
miR-34b-5p
miR-34c-5p
miR-1983
miR-483-5p
miR-185-5p
miR-21a-5p
miR-298-5p
miR-31-5p
log2(ratio) (mdx vs C57)
log2(mean normalized counts)
miR-34b-5p
miR-31-5p
miR-483-3p
miR-34c-5p
miR-21a-5p
Differentially expressed (adjusted p<0.05)
C57
mdx
PPMO
Diaphragm
Gastrocnemius
Soleus
TA
Row Z-score
3.0
0.0
-3.0
miR-185-5p
miR-215-5p
miR-673-3p
miR-540-5p
miR-493-5p
miR-666-5p
miR-338-5p
miR-382-3p
miR-409-5p
miR-434-3p
miR-337-5p
miR-21a-5p
miR-532-5p
miR-31-5p
miR-455-5p
miR-501-3p
miR-221-3p
miR-7688-5p
miR-193b-3p
miR-296-3p
miR-1983
miR-34b-5p
miR-483-5p
miR-298-5p
miR-483-3p
miR-34c-5p
miR-296-5p
miR-362-5p
miR-222-3p
miR-214-3p
miR-652-3p
miR-362-3p
miR-532-3p

Figure S12

Differential miRNA expression in pooled muscle samples

Counts data for all four muscles were pooled to increase sample sizes and differential expression analysis performed to identify statistically significant changes (adjusted $p < 0.05$) between *mdx* and C57 muscle. Differential expressed miRNAs in *mdx* relative to wild-type controls as visualized by (A) volcano plot, and (B) MA plot. Statistically significant changes are highlighted in red and blue (for elevated and reduced levels in *mdx* muscle respectively). Labels are shown for miRNAs of interest. (C) Heatmap of significantly changed miRNAs in *mdx* serum, showing the effect of PPMO treatment on circulating miRNA levels. The labels for miRNAs of interest are highlighted in red. Scale bars show mean-centered $\log_2$ normalized counts (row Z-score) where red and blue indicate higher and lower than mean abundance respectively.

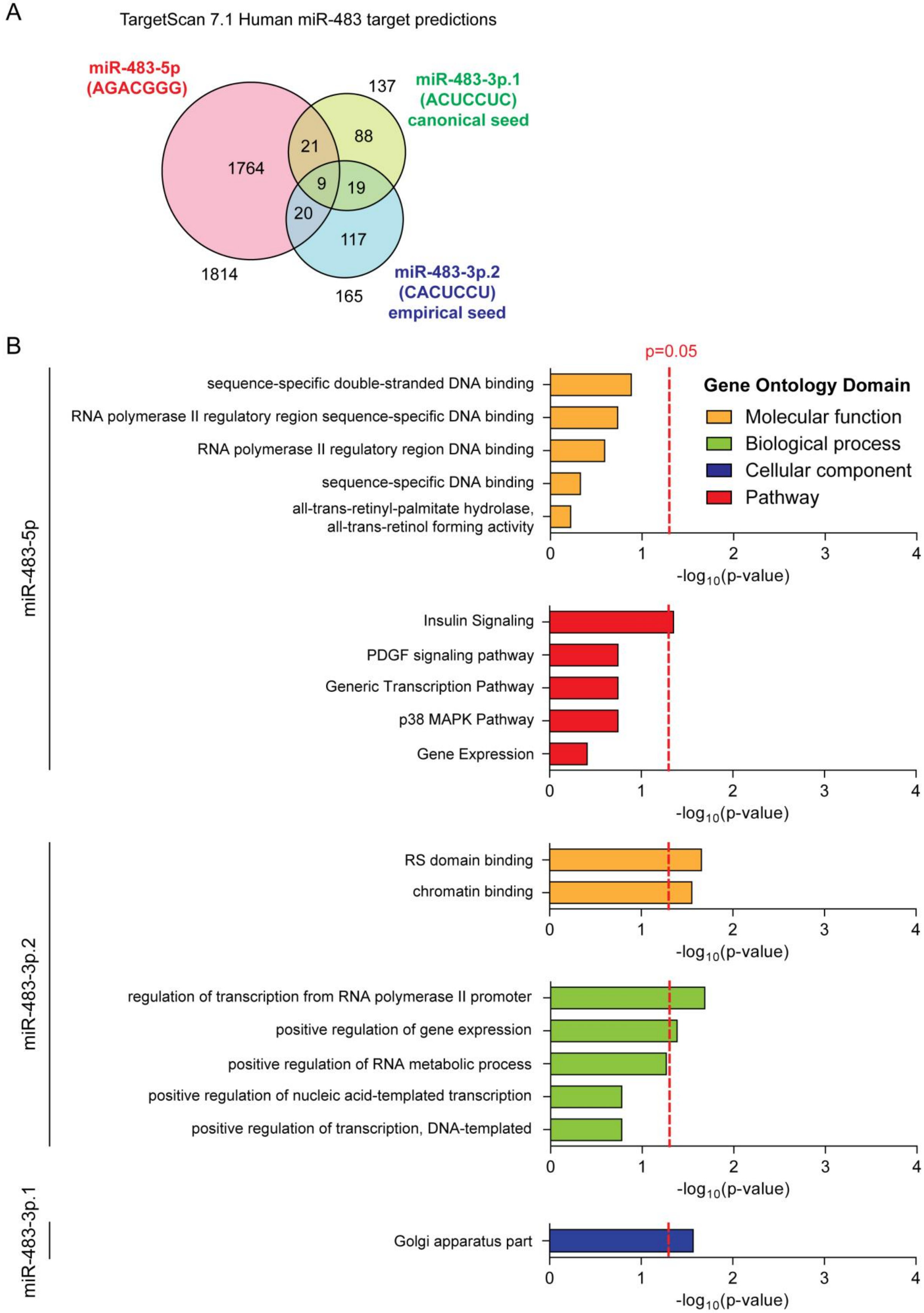
A
TargetScan 7.1 Human miR-483 target predictions
miR-483-5p
(AGACGGG)
137
miR-483-3p.1
(ACUCCUC)
canonical seed
88
21
1764
9
19
20
117
1814
miR-483-3p.2
(CACUCCU)
empirical seed
165
B
p=0.05
Gene Ontology Domain
Molecular function
Biological process
Cellular component
Pathway
miR-483-5p
sequence-specific double-stranded DNA binding
RNA polymerase II regulatory region sequence-specific DNA binding
RNA polymerase II regulatory region DNA binding
sequence-specific DNA binding
all-trans-retinyl-palmitate hydrolase,
all-trans-retinol forming activity
-log10(p-value)
Insulin Signaling
PDGF signaling pathway
Generic Transcription Pathway
p38 MAPK Pathway
Gene Expression
-log10(p-value)
miR-483-3p.2
RS domain binding
chromatin binding
-log10(p-value)
regulation of transcription from RNA polymerase II promoter
positive regulation of gene expression
positive regulation of RNA metabolic process
positive regulation of nucleic acid-templated transcription
positive regulation of transcription, DNA-templated
-log10(p-value)
miR-483-3p.1
Golgi apparatus part
-log10(p-value)

Figure S13

***In silico* Analysis of predicted miR-483 targets**

(A) Venn diagram of predicted mRNA targets for human miR-483-5p and miR-483-3p (both canonical and empirically-determined seed sequences) generated using the TargetScan algorithm. (B) Gene ontology terms enriched in the lists of predicted target mRNAs for each miRNA. Benjamini-Hochberg corrected p-values are reported.

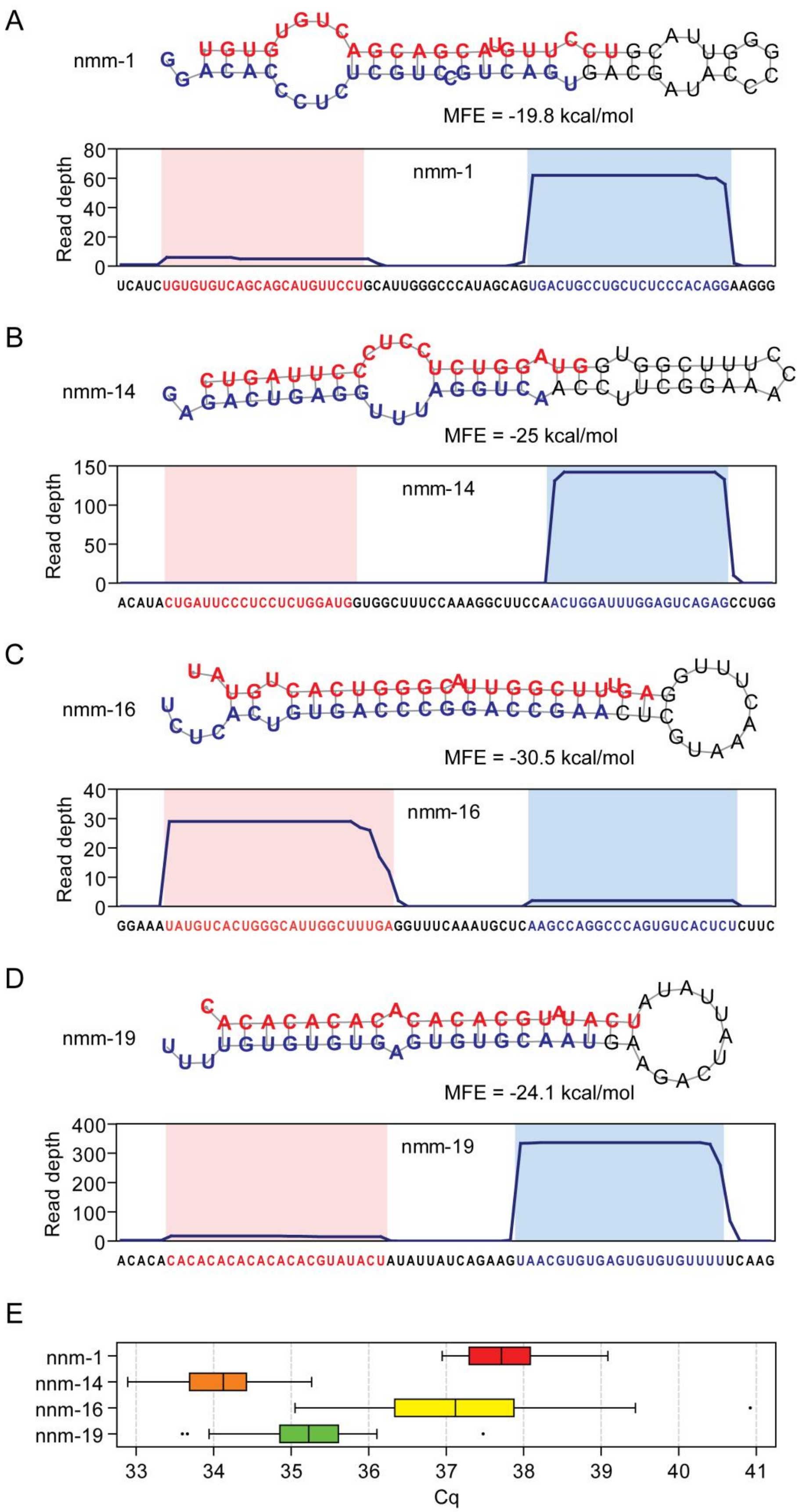

A
nmm-1
MFE = -19.8 kcal/mol
Read depth
0
20
40
60
80
UCAUCUGUGUGUCAGCAGCAUGUUCCUGCAUUGGGCCCAUAGCAGUGACUGCCUGCUCUCCCACAGGAAGGG
B
nmm-14
MFE = -25 kcal/mol
Read depth
0
50
100
150
ACAUACUGAUUCCCUCCUCUGGAUGGUGGCUUUCCAAAGGCUUCCAACUGGAUUUGGAGUCAGAGCCUGG
C
nmm-16
MFE = -30.5 kcal/mol
Read depth
0
10
20
30
40
GGAAAUAUGUCACUGGGCAUUGGCUUUGAGGUUUCAAAUGCUCAAGCCAGGCCCAGUGUCACUCUCUUC
D
nmm-19
MFE = -24.1 kcal/mol
Read depth
0
100
200
300
400
ACACACACACACACACACGUAUACUAUAUUAUCAGAAGUAACGUGUGAGUGUGUGUUUUUCAAG
E
nnm-1
nnm-14
nnm-16
nnm-19
33
34
35
36
37
38
39
40
41
Cq

Figure S14

Identification of novel miRNAs in muscle

RNA folding structures of precursor hairpins and miRNA signature plots for the novel miRNAs (A) nmm-1, (B) nmm-14, (C) nmm-16, and (D) nmm-19. Major and minor miRNA species are highlighted in red and blue respectively. Minimum Free Energy (MFE) values are indicated. (E) Novel miRNAs were detected in all muscle samples assayed (n = 36) and Cq values visualised by Tukey box plot.

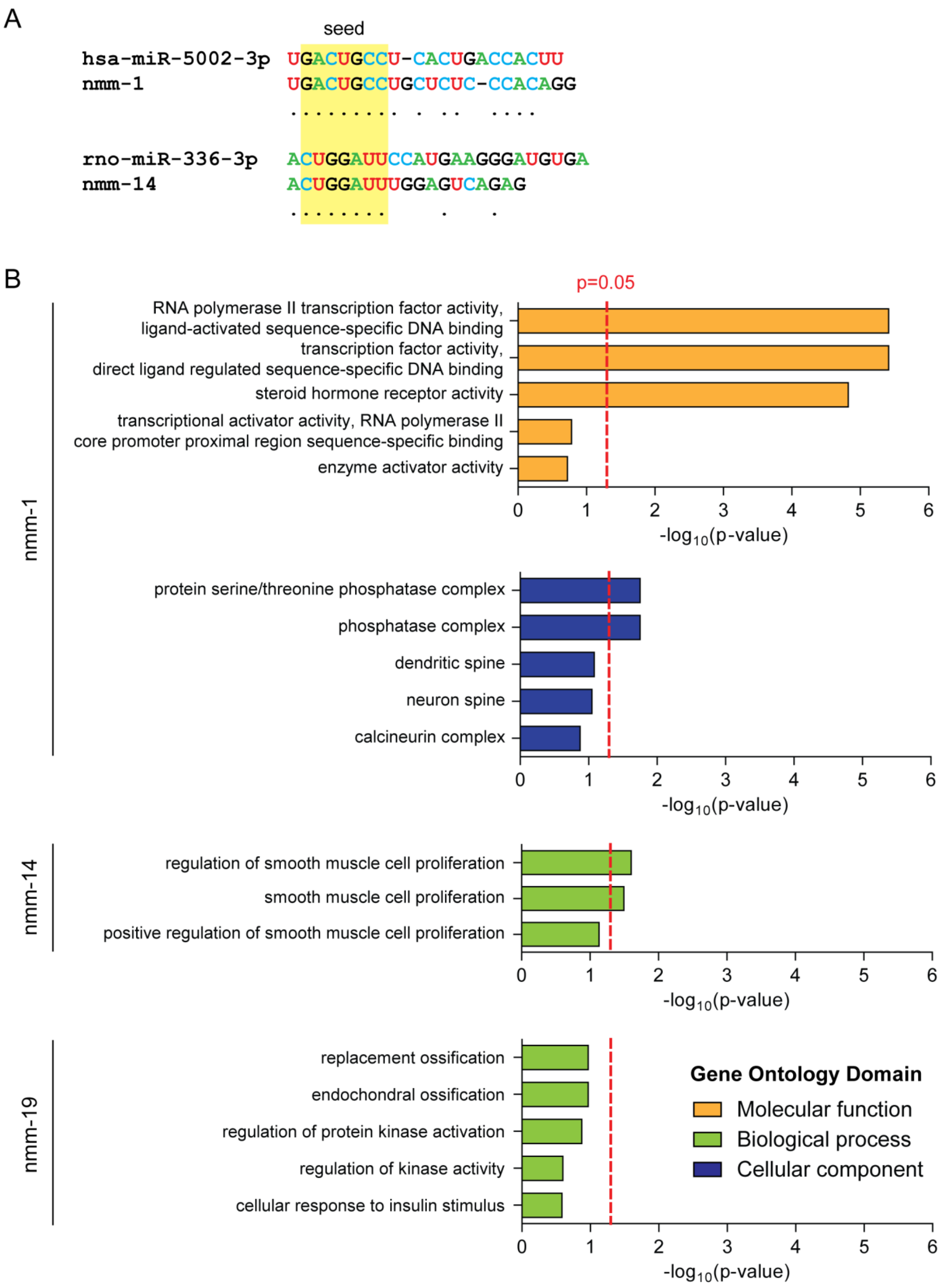
A
seed
hsa-miR-5002-3p UGACUGCCU-CACUGACCACUU
nmm-1 UGACUGCCUGCUCUC-CCACAGG
rno-miR-336-3p ACUGGAUUCCAUGAAGGGAUGUGA
nmm-14 ACUGGAUUUGGAGUCAGAG
B
p=0.05
nmm-1
RNA polymerase II transcription factor activity, ligand-activated sequence-specific DNA binding
transcription factor activity, direct ligand regulated sequence-specific DNA binding
steroid hormone receptor activity
transcriptional activator activity, RNA polymerase II core promoter proximal region sequence-specific binding
enzyme activator activity
protein serine/threonine phosphatase complex
phosphatase complex
dendritic spine
neuron spine
calcineurin complex
nmm-14
regulation of smooth muscle cell proliferation
smooth muscle cell proliferation
positive regulation of smooth muscle cell proliferation
nmm-19
replacement ossification
endochondral ossification
regulation of protein kinase activation
regulation of kinase activity
cellular response to insulin stimulus
0 1 2 3 4 5 6
-log10(p-value)
Gene Ontology Domain
Molecular function
Biological process
Cellular component

Figure S15

Predicted targets for novel miRNAs

(A) The novel miRNAs nmm-1 and nmm-14 share seed sequences with hsa-miR-5002-3p and rno-miR-336-3p respectively. (B) Predicted target mRNAs for novel miRNAs were generated using the miRDB resource. Gene ontology terms enriched in the lists of predicted target mRNAs for each novel miRNA. Benjamini-Hochberg corrected p-values are reported.

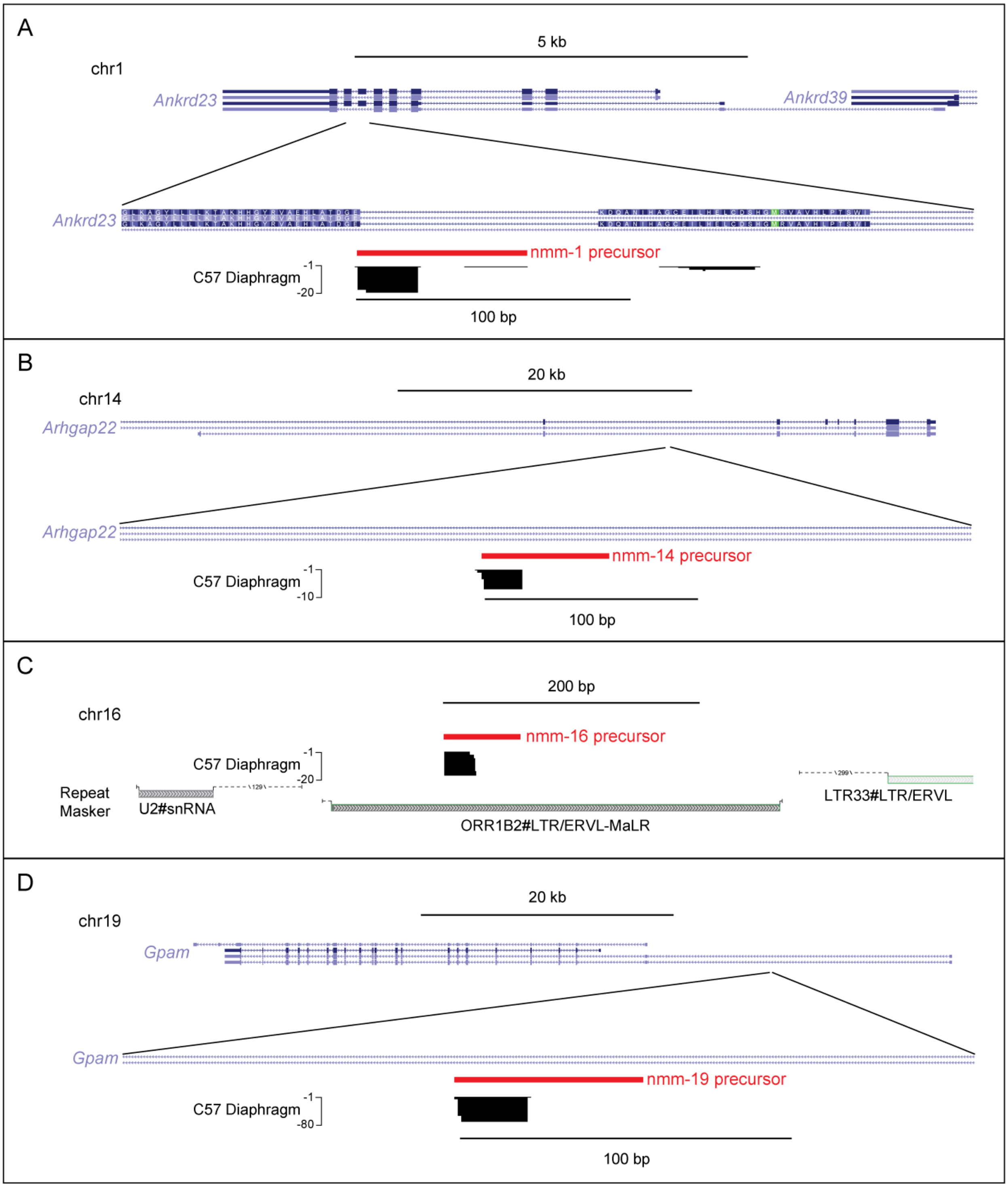
A
5 kb
chr1
Ankrd23
Ankrd39
Ankrd23
nmm-1 precursor
C57 Diaphragm
-1
-20
100 bp
B
20 kb
chr14
Arhgap22
Arhgap22
nmm-14 precursor
C57 Diaphragm
-1
-10
100 bp
C
200 bp
chr16
nmm-16 precursor
C57 Diaphragm
-1
-20
Repeat Masker
U2#snRNA
ORR1B2#LTR/ERVL-MaLR
LTR33#LTR/ERVL
D
20 kb
chr19
Gpam
Gpam
nmm-19 precursor
C57 Diaphragm
-1
-80
100 bp

Figure S16

Genomic location of novel miRNA precursors

Locations of novel miRNA precursors (red bars) are shown for (A) nmm-1, (B) nmm-14, (C) nmm-16, and (D) nmm-19. Representative sequencing read density is shown for one library (C57 Diaphragm). nmm-1 and nmm-19 reside within introns of the protein coding genes *Ankrd23* and *Gpam* respectively. nmm-14 is antisense to an intron of *Arhgap22*. Conversely, nmm-16 appears to be intergenic, and originates from an integrated LTR element (covered by the RepeatMasker in the UCSC Genome Browser).

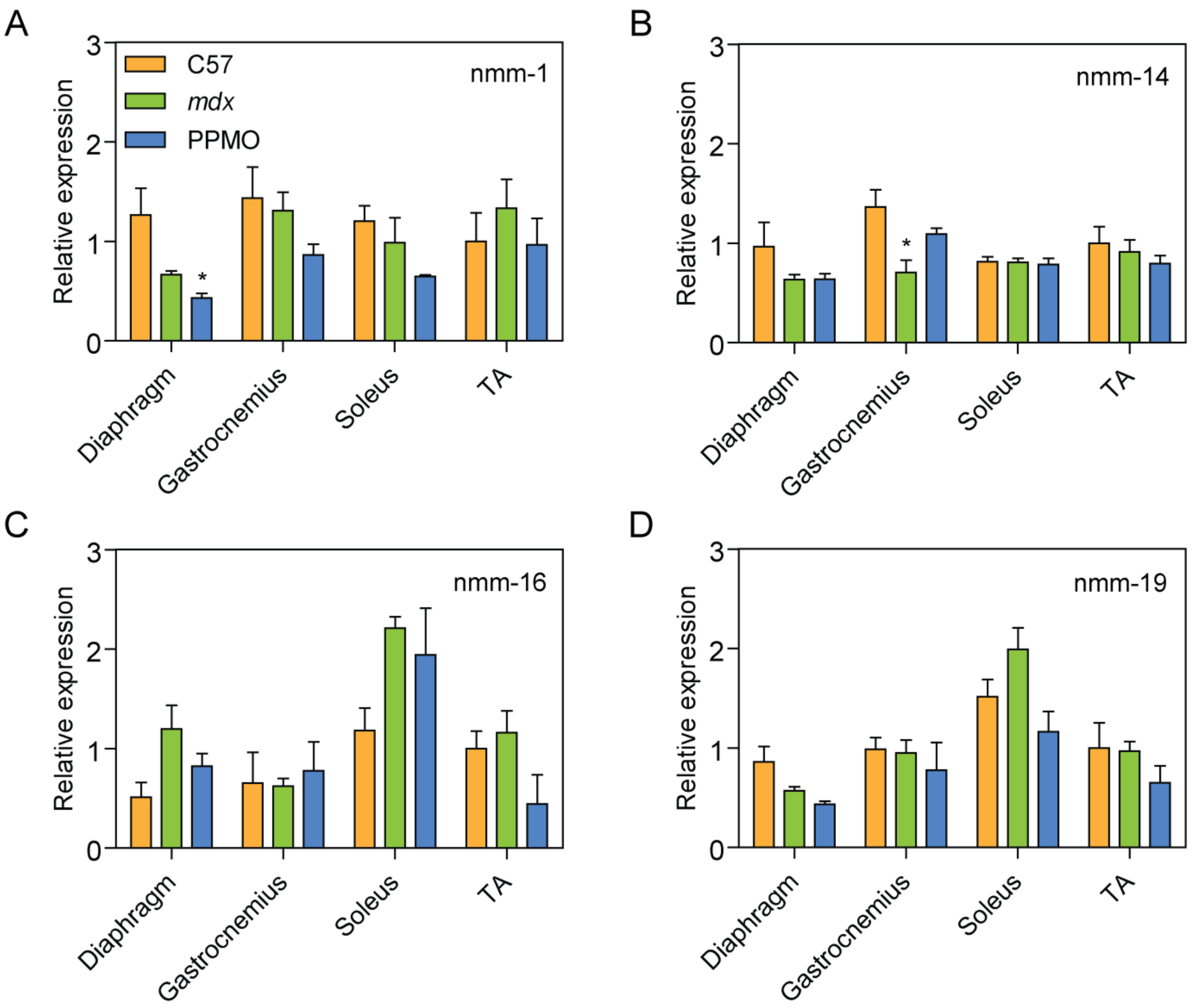

Figure S17

Expression of novel miRNAs in normal and dystrophic muscle

Novel miRNA expression was measured by qRT-PCR for C57, *mdx* and PPMO-treated samples in (A) Diaphragm, (B) Gastrocnemius, (C) Soleus and (D) TA. Novel miRNA levels were normalized to miR-16-5p expression and all expression ratios scaled such that the mean of the C57 TA group was returned to one. Values are mean+SEM, $n = 3$, $^*p < 0.05$, one-way ANOVA with Bonferroni *post hoc* test. All statistical comparisons indicated are relative to the C57 control group.

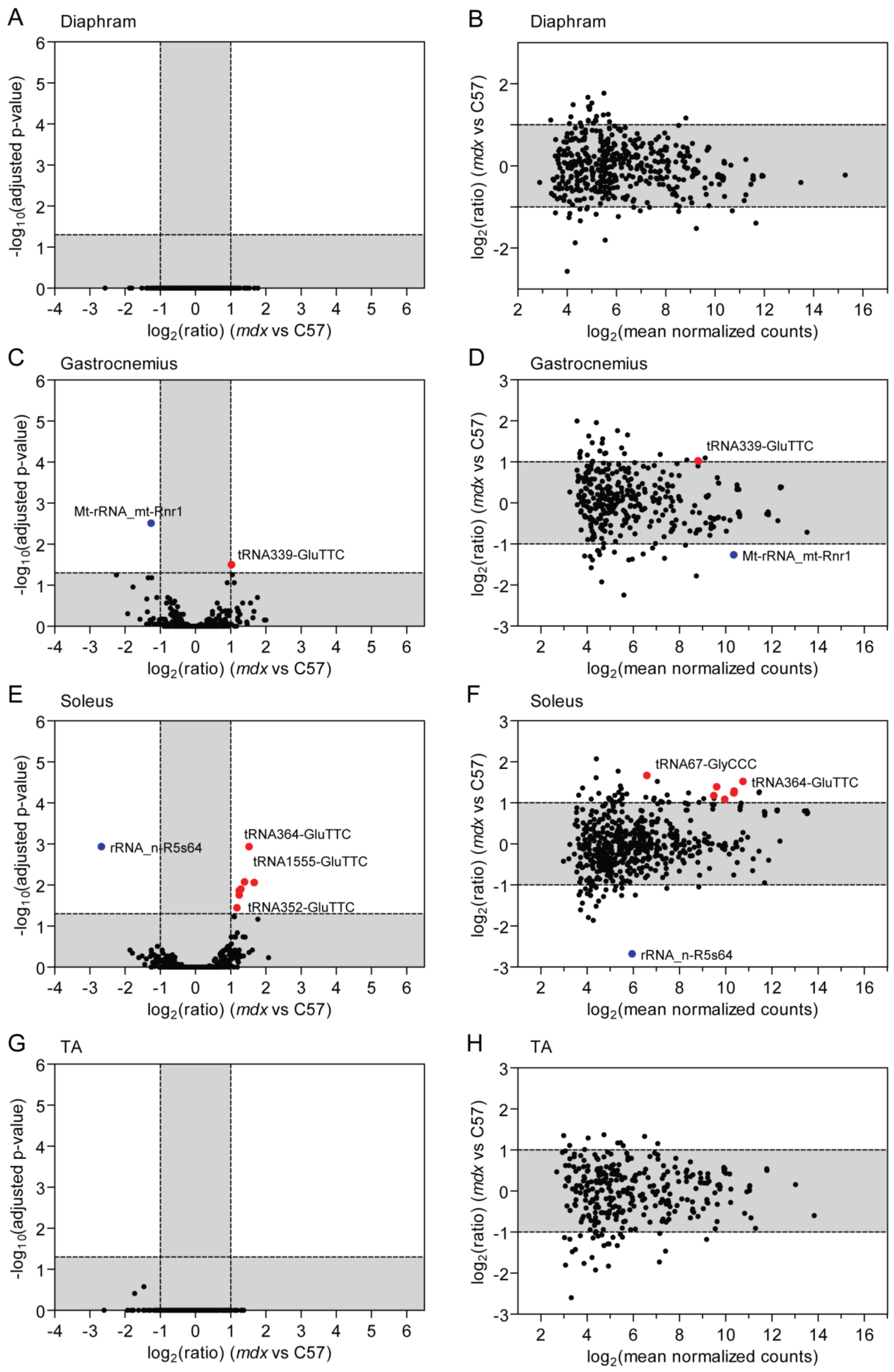

A
Diaphram
-log10(adjusted p-value)
log2(ratio) (mdx vs C57)
B
Diaphram
log2(ratio) (mdx vs C57)
log2(mean normalized counts)
C
Gastrocnemius
Mt-rRNA_mt-Rnr1
tRNA339-GluTTC
-log10(adjusted p-value)
log2(ratio) (mdx vs C57)
D
Gastrocnemius
tRNA339-GluTTC
Mt-rRNA_mt-Rnr1
log2(ratio) (mdx vs C57)
log2(mean normalized counts)
E
Soleus
rRNA_n-R5s64
tRNA364-GluTTC
tRNA1555-GluTTC
tRNA352-GluTTC
-log10(adjusted p-value)
log2(ratio) (mdx vs C57)
F
Soleus
tRNA67-GlyCCC
tRNA364-GluTTC
rRNA_n-R5s64
log2(ratio) (mdx vs C57)
log2(mean normalized counts)
G
TA
-log10(adjusted p-value)
log2(ratio) (mdx vs C57)
H
TA
log2(ratio) (mdx vs C57)
log2(mean normalized counts)

Figure S18

Differential expression of non-miRNA ncRNA species in dystrophic muscle

Differential expression of non-miRNA sRNAs in *mdx* muscle relative to wild-type controls as visualized by volcano and MA plots for (A,B) diaphragm, (C,D) gastrocnemius, (E,F) soleus, and (G,H) TA. Statistically significant changes are highlighted in red and blue (for elevated and reduced levels in *mdx* serum respectively). Labels are shown for ncRNAs of interest. For Diaphragm and TA there were no significant differential ncRNA expression calls.

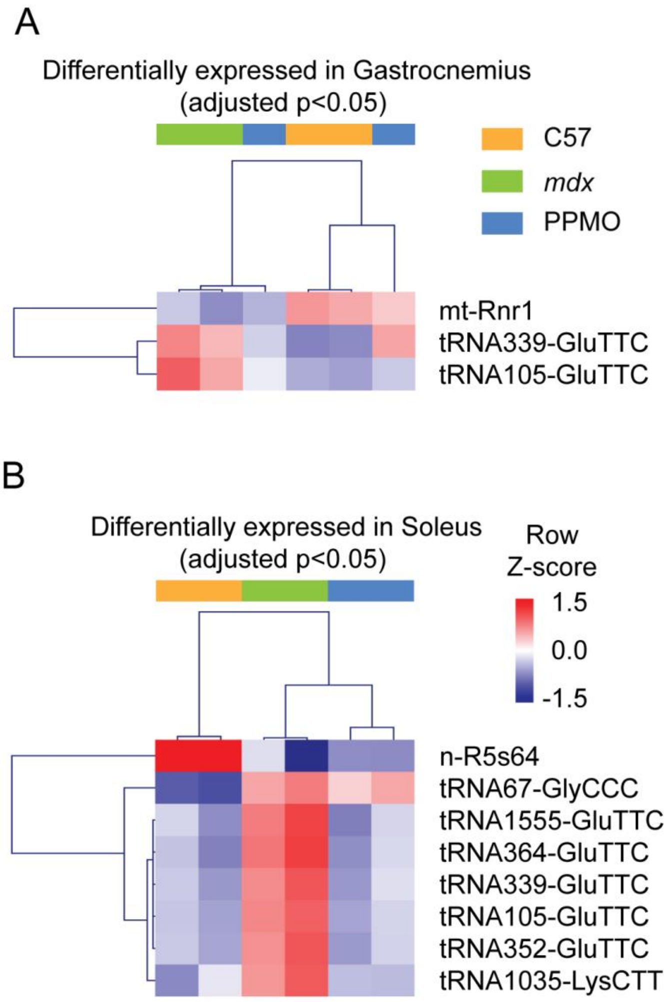

Figure S19

Heatmap analysis of non-coding RNA expression after PPMO treatment in muscle

Statistically significant (adjusted $p < 0.05$) non-miRNA sRNA expression ratios were visualized by hierarchical clustering analysis and heatmaps generated comparing wild-type (C57), dystrophic (*mdx*), and PPMO-treated *mdx* mice in (A) gastrocnemius, and (B) soleus muscles. (No statistically significant non-miRNA ncRNAs were identified in diaphragm or TA). Scale bars show mean-centered, $\log_2$ normalized counts (row Z-score) where red and blue indicate higher and lower than mean expression respectively.

ID	Genomic location (mm10)	Mature miRNA sequence (miRNA with conserved seed)	Mean miRDeep2 score	Mean read count	Overlapping gene
**nmm-1**	chr1:36531820-36531882	UGACUGCCUGCUCUCCCACAGG (hsa-miR-5002-3p)	13	13.6	*Ankrd23* (intronic)
**nmm-14**	chr14:33351716-33351776	ACUGGAUUUGGAGUCAGAG (rno-miR-336-3p)	4.8	20.1	*Arhgap22* (antisense)
**nmm-16**	chr16:57610785-57610845	UAUGUCACUGGGCAUUGGCUUUG	8.8	15.5	Repeat (intergenic)
**nmm-19**	chr19:55112918-55112974	UAACGUGUGAGUGUGUGUUUU	34.4	70.2	*Gpam* (intronic)

Table S1
Properties of novel miRNAs

	Assay ID
**Small RNA TaqMan**	
**miR-1a-3p**	002222
**miR-16-5p**	000391
**miR-21a-5p**	000397
**miR-31-5p**	000185
**miR-34c-5p**	000428
**miR-126a-3p**	002228
**miR-133a-3p**	002246
**miR-206a-3p**	000510
**mmu-miR-483-3p**	002560
**hsa-miR-483-5p**	002338
**hsa-miR-483-3p**	002339
**cel-miR-39**	000200
**miScript Assays**	
**Ce-miR-39-1**	MS00019789
**Ce-miR-238-1**	MS00019439
**Mm_miR-133a_2**	MS00032305
**piR_000620**	MSC0075706
**piR_000935**	MSC0075707

Table S2
List of qRT-PCR Assays used in this study
Product IDs for Small RNA qRT-PCR (Life Technologies) and miScript assays (Qiagen).

	Target Sequence
**piRNAs**	
**piR_000620**	CGGGCCGCCGGUGAAAUACCACUAC
**piR_000935**	GGCUGGUCCGAAGGUAGUGAGUUAUCUCAAU
**Novel miRNAs**	
**nmm-1**	UGACUGCCUGCUCUCCCACAGG
**nmm-14**	ACUGGAUUUGGAGUCAGAG
**nmm-16**	UAUGUCACUGGGCAUUGGCUUUGA
**nmm-19**	UAACGUGUGAGUGUGUGUUUU

Table S3
Target Sequences for Custom Small RNA TaqMan Assays
All sequences are 5′ to 3′.

Supplemental Materials and Methods

Analysis of Exon Skipping by qRT-PCR

Total RNA (1 µg) was reverse transcribed using the High Capacity cDNA Kit (Life Technologies, Paisley, UK) according to manufacturer's instructions. Levels of *Dmd* exon 23 skipping were determined by probe based qRT-PCR using a FAM-assay spanning the exon 20-21 boundary (total *Dmd* transcript expression) and a HEX-assay spanning the exon 23-24 boundary (unskipped *Dmd* transcript expression) (Assay IDs: Mm.PT.47.9564450 and Mm.PT.47.7668824 respectively, Integrated DNA Technologies, Leuven, Belgium). qPCR was performed on a StepOne Plus real-time PCR Thermocycler using TaqMan Gene Expression Master Mix (both Life Technologies) and 25 ng of cDNA template per reaction. The percentage of *Dmd* exon 23 skipping was determined by calculating (1 – the ratio of unskipped:total *Dmd* transcripts) × 100%.

Dystrophin Western Blot

To quantify dystrophin protein expression, 8 µm cryosections were prepared from the mid-belly of TA and gastrocnemius muscle and samples lysed in buffer (50 mM Tris pH 8, 150 mM NaCl, 1% NP-40, 0.5% sodium deoxycholate, 10% sodium dodecyl sulphate, and protease inhibitors). Due to limitations in the amount of sample material, dystrophin mRNA skipping was analyzed only in soleus and diagram. Protein lysates were incubated at 100°C for 3 minutes and then centrifuged at 14,000 *g* for 10 minutes at 4°C to pellet debris. Equal amounts of protein lysate (40 µg) were separated on a 3-8% Tris-Acetate gel (Life Technologies), electrotransferred to a Polyvinylidene fluoride (PVDF) membrane and probed with monoclonal anti-dystrophin (1:200, NCL-DYS1, Leica Biosystems, Lincoln, NE) and anti-vinculin (loading control, 1:100,000, hVIN-1, Sigma, Dorset, UK) primary antibodies as previously described [1]. Secondary antibody IRDye 800CW goat anti-mouse was used at a dilution of 1:10,000 (LiCOR Biosystems). Fluorescence was detected and quantified using the Odyssey imaging system. Vinculin was used as a loading control. To quantify dystrophin expression, the dystrophin to vinculin ratio for each PPMO-treated sample were compared with a dilution of series of C57Bl/10 lysate (diluted in *mdx* lysate to maintain consistent vinculin expression levels between standards) as described previously [2].

Determination of piRNA 3′ Terminus Identity

Unlike other classes of small RNAs, piRNAs are modified at their 3′ terminus with a 2′-O-methyl group, similar to plant miRNAs. Consequently, the miScript Plant RT Kit (Qiagen, Fredrick, MD, USA) was utilized to determine the methylation status of the putative piRNA 3′ termini in combination with an oxidation and β-elimination reaction. This reaction converts the unmodified 2′ hydroxyl groups of the ribose sugar into a di-aldehyde for animal miRNAs [3] which inhibits the ligation of the linker during the RT, and so unmodified small RNAs become invisible to qRT-PCR after oxidation/β-elimination. Conversely, piRNAs are protected from conversion as a consequence of their 2′-O-methylation.

To determine the identity of putative piRNA 3′ termini, RNA was extracted from *mdx* TA muscle and 1 µl each of 5 pM cel-miR-39 and cel-miR-238-2′OMe were added as exogenous controls. RNA was subjected to periodate oxidation and β-elimination as described previously [3] with slight modifications. 50 µl reaction mix containing 6 µg total RNA and 10 mM $NaIO_4$ was incubated at 0°C for 40 minutes in the dark. Next, RNA was purified by ethanol purification and an equal volume of 2M Lys-HCl added. Samples were then incubated at 45°C for 90 minutes. After a second ethanol precipitation, 400 ng of reaction product was reverse transcribed according to the manufacturer's instructions. cDNA was generated from untreated RNA in parallel as a control. All samples were subsequently quantified by qPCR utilizing miScript Primer Assays (**Table S3**) and miScript SYBR Green PCR Kit (both Qiagen, Manchester, UK).

miRNA Bioinformatics

Target prediction for known miRNAs (i.e. hsa-miR-483) was performed using TargetScan v7.1 [4]. (Predicted targets are listed in **Data S2**). Target prediction for novel miRNAs were performed using the miRDB resource [5]. (Predicted targets are listed in **Data S3**). Gene list enrichment of novel-miRNA target mRNAs was performed using ToppFun [6]. RNA folding structures and Minimum Free Energy values were calculated using the Vienna RNAfold resource [7]. miRNA signature plots were generated using custom R scripts.

Small RNA-seq Bioinformatics and Statistics

Quality Control

The FASTQC package (v0.11.3) was used to assess the quality of sequenced libraries (raw FASTQ files) using the following command:

```
fastqc input.fastq
```

Adaptor Removal

FASTQ file output from sequencing runs uniformly contained 51 nucleotide reads. Considering that the vast majority of small RNA species in these samples were expected to be in the 20-30 nt range, the majority of reads contained 3′ adaptor sequences. Adaptors were trimmed using Cutadapt (v1.10) [8] so as to exclude reads containing the adaptor sequence, and those reads that where <17 nt in length using the following generic command:

```
cutadapt -a AGATCGGAAGAGCACACGTCT  --minimum-length 17 --trimmed-only -o
adaptor-trimmed_inputfile.fastq inputfile.fastq
```

`-a`	Specifies 3′ adaptor removal
`-- minimum-length 17`	Discards reads less than 17 nucleotides in length

Read length distributions were obtained using the following command:

```
awk 'NR%4 == 2 {lengths[length($0)]++} END {for (l in lengths) {print l,
lengths[l]}}' adaptor-trimmed_inputfile.fastq
```

Indexing a Reference Genome File

A reference *Mus musculus* genome (mm10) was built using FASTA files for each chromosome (downloaded from https://ccb.jhu.edu/software/tophat/index.shtml) using the bowtie-build (v0.1.7) command:

```
bowtie-build -f
chr1.fa,chr2.fa,chr3.fa,chr4.fa,chr5.fa,chr6.fa,chr7.fa,chr8.fa,chr9.fa,chr10
.fa,chr11.fa,chr12.fa,chr13.fa,chr14.fa,chr15.fa,chr16.fa,chr17.fa,chr18.fa,c
hr19.fa,chrM.fa,chrX.fa,chrY.fa &
```

`-f` Specifies FASTA format

miRNA Mapping and Counting

Sequences of mature and hairpin precursor miRNAs were downloaded from miRBase (version 21) [9] and species-specific miRNA subsets extracted using the following commands within the miRDeep2 package (v0.0.8) [10]:

```
extract_miRNAs.pl mature.fa mmu >mature_mmu.fa
extract_miRNAs.pl hairpin.fa mmu >hairpin_mmu.fa
extract_miRNAs.pl mature.fa rno,hsa >mature_not_mmu.fa
```

Sequence reads were mapped to an indexed genome using the mapper miRDeep2 module:

```
mapper.pl adaptor-trimmed_inputfile.fastq -e -p reference-genome -s
processed_reads.fa -t mapped_reads.arf -h -m -i –j -v
```

`-e`	Specifies input file is in .fastq format
`-p`	Specifies mapping to a genome that has been indexed using `bowtie-build`
`-t`	Print read mappings to specified .arf file

`-h`	Parse to fasta format
`-m`	Collapse reads
`-i`	Converts RNA to DNA alphabet
`-j`	Removes all entries that have a sequence that contains letters other than a,c,g,t,u,n,A,C,G,T,U,N
`-v`	Outputs progress report

miRDeep2 analysis was performed using the following generic command:

```
miRDeep2.pl processed_reads.fa genome.fa mapped_reads.arf mature.fa
mature_non.fa hairpin.fa -z [tag] 2 > report.log
```

`-P`	Specifies use of latest miRbase nomenclature
`-z`	Defines sample output tag

Mapping of non-coding RNA Species

Sequencing reads were mapped to the mouse genome using Bowtie (v 0.12.7) [11] using the following generic command:

```
bowtie -p 48 -v2  -m20 --best --strata -S --chunkmbs 200 $BOWTIE_IND
input.fastq output.sam
```

`-p 48`	Specifies multi-threading
`-m20`	Discards all reads that align with the index more than 20 times
`--best --strata`	Reports only the best read for multi-mapping reads
`--chunkmbs 200`	Increases available memory usage to 200 mb
`-S`	Indicates output in .sam format
`BOWTIE_IND`	Path to reference genome indexed using `bowtie-build`

Alignment files were sorted using the SAMtools package (v1.3) [12]:

```
$SAMTOOLS sort input.sam -o output_sorted.sam
```

`SAMTOOLS`	Path to SAMtools (v1.3)
`-o`	Specifies output file

Counting Reads for non-coding RNA Species

A reference .gtf file was generated by extracting the coordinates of miRNAs, rRNAs, snRNA, snoRNA, scaRNA, mtRNA from the *Mus musculus* GRCm38 (mm10) annotation file available on the Ensembl website (http://www.ensembl.org/info/data/ftp/index.html) using the `grep` command:

```
grep "snoRNA" Mus_musculus.GRCm38.84.gtf >snoRNA.gtf
grep "Mt_rRNA" Mus_musculus.GRCm38.84.gtf >Mt_rRNA.gtf
grep "Mt_tRNA" Mus_musculus.GRCm38.84.gtf >Mt_tRNA.gtf
grep "rRNA" Mus_musculus.GRCm38.84.gtf >rRNA.gtf
grep "snRNA" Mus_musculus.GRCm38.84.gtf >snRNA.gtf
grep "sRNA" Mus_musculus.GRCm38.84.gtf >sRNA.gtf
grep "scaRNA" Mus_musculus.GRCm38.84.gtf >scaRNA.gtf
grep "miRNA" Mus_musculus.GRCm38.84.gtf >miRNA.gtf
```

Genome coordinates for tRNAs were extracted from the UCSC table browser (https://genome.ucsc.edu/cgi-bin/hgTables) and piRNA annotation was obtained from the piRNABank [13] and converted to the GRCm38.84

coordinate system using the USCS liftOver tool. Individual .gtf files were combined into a single annotation file utilizing the `cat` command:

```
cat piRNA.gtf tRNA.gtf miRNA.gtf snRNA.gtf snoRNA.gtf scaRNA.gtf Mt_tRNA.gtf
rRNA.gtf Mt_rRNA.gtf > ncRNA_ensemble.gtf
```

Chromosome label format was adjusted to the correct format (i.e. chr1) using the following command:

```
sed -e 's/^\([0-9XY][0-9]*\)/chr\1/; s/^MT/chrM/;' nc_miRNA.gtf >
nc_miRNA_chr.gtf
```

Mapped and sorted reads were counted with the HTSeq package (v0.6.1) [14] using the following command:

```
python $HTSEQ -m union -s yes input.sam $GENEREF  > output_counts_ncRNA.txt
```

`HTSEQ`	Path to htseq-count program
`GENEREF`	Path to .gtf file
`-m union`	Counting mode setting
`-s yes`	Specifies stranded library

Data Visualization

The SAMtools package (v1.3) [12] was used to convert sorted .sam files to .bam format for each alignment:

```
$SAMTOOLS view -bS input_sorted.sam output_sorted.bam
```

`SAMTOOLS`	Path to SAMtools (v1.3)
`-bS`	Defines input format as SAM and output as BAM

Index files for the bam outputs (.bai) were generated using the following generic command:

```
$SAMTOOLS index input_sorted.bam
```

`SAMTOOLS`	Path to SAMtools (v1.3)

Stranded bigwig files were generated using bam2wig.py (v2.6.3) in order to visualize sequencing read density using the following generic command:

```
$PYTHON $BAMtoBW_BIN -s $CHROMSIZE -i input_sorted.bam -o $OUTPUT -d '++,--'
```

`PYTHON`	Path to python (v2.7)
`BAMtoBW_BIN`	Path to bam2wig.py
`CHROMSIZE`	Path to text file containing chromosome sizes
`OUTPUT`	Path for output files
`-s`	Defines chromosome size file
`-i`	Defines input file
`-o`	Defines output prefix
`-d '++,--'`	Indicates strandedness rule

Filtering of Novel Candidate miRNAs

To identify potential novel miRNAs in our datasets, mirDeep2 output for all *mdx* and wild-type tissue samples were filtered to retain candidates with a true positive rate ≥ 1 and a miRDeep2 score ≥ 1. Candidates were discarded if they (a) were detected in only a single library, (b) had non-significant Randfold p-values (i.e. energetically

unfavorable hairpin structures), and (c) had Rfam alerts (i.e. high similarity with other known RNA species). After filtering, miRNA signature plots were visually inspected for each of the remaining 11 potential novel miRNAs. Of these, four candidate miRNAs were selected for further analysis based on how well sequencing reads mapped to each hairpin structure [15].

Supplemental References

[1] Roberts TC, Godfrey C, McClorey G, Vader P, Briggs D, Gardiner C, et al. Extracellular microRNAs are dynamic non-vesicular biomarkers of muscle turnover. Nucl Acids Res 2013;41:9500–13. doi:10.1093/nar/gkt724.

[2] Godfrey C, Muses S, McClorey G, Wells KE, Coursindel T, Terry RL, et al. How much dystrophin is enough: the physiological consequences of different levels of dystrophin in the mdx mouse. Hum Mol Genet 2015;24:4225–37. doi:10.1093/hmg/ddv155.

[3] Ohara T, Sakaguchi Y, Suzuki T, Ueda H, Miyauchi K, Suzuki T. The 3′ termini of mouse Piwi-interacting RNAs are 2′-O-methylated. Nat Struct Mol Biol 2007;14:349–50. doi:10.1038/nsmb1220.

[4] Agarwal V, Bell GW, Nam J-W, Bartel DP. Predicting effective microRNA target sites in mammalian mRNAs. Elife 2015;4. doi:10.7554/eLife.05005.

[5] Wong N, Wang X. miRDB: an online resource for microRNA target prediction and functional annotations. Nucleic Acids Res 2015;43:D146-152. doi:10.1093/nar/gku1104.

[6] Chen J, Bardes EE, Aronow BJ, Jegga AG. ToppGene Suite for gene list enrichment analysis and candidate gene prioritization. Nucleic Acids Res 2009;37:W305-311. doi:10.1093/nar/gkp427.

[7] Lorenz R, Bernhart SH, Höner Zu Siederdissen C, Tafer H, Flamm C, Stadler PF, et al. ViennaRNA Package 2.0. Algorithms Mol Biol 2011;6:26. doi:10.1186/1748-7188-6-26.

[8] Martin M. Cutadapt removes adapter sequences from high-throughput sequencing reads. EMBnetJournal 2011;17:10–2. doi:10.14806/ej.17.1.200.

[9] Griffiths-Jones S, Saini HK, van Dongen S, Enright AJ. miRBase: tools for microRNA genomics. Nucleic Acids Res 2008;36:D154-158. doi:10.1093/nar/gkm952.

[10] Friedländer MR, Mackowiak SD, Li N, Chen W, Rajewsky N. miRDeep2 accurately identifies known and hundreds of novel microRNA genes in seven animal clades. Nucleic Acids Res 2012;40:37–52. doi:10.1093/nar/gkr688.

[11] Langmead B, Trapnell C, Pop M, Salzberg SL. Ultrafast and memory-efficient alignment of short DNA sequences to the human genome. Genome Biol 2009;10:R25. doi:10.1186/gb-2009-10-3-r25.

[12] Li H, Handsaker B, Wysoker A, Fennell T, Ruan J, Homer N, et al. The Sequence Alignment/Map format and SAMtools. Bioinformatics 2009;25:2078–9. doi:10.1093/bioinformatics/btp352.

[13] Lakshmi SS, Agrawal S. piRNABank: a web resource on classified and clustered Piwi-interacting RNAs. Nucl Acids Res 2008;36:D173–7. doi:10.1093/nar/gkm696.

[14] Anders S, Pyl PT, Huber W. HTSeq--a Python framework to work with high-throughput sequencing data. Bioinformatics 2015;31:166–9. doi:10.1093/bioinformatics/btu638.

[15] Kang W, Friedländer MR. Computational Prediction of miRNA Genes from Small RNA Sequencing Data. Front Bioeng Biotechnol 2015;3:7. doi:10.3389/fbioe.2015.00007.
